# Cytotoxic and Antibacterial Cyclodepsipeptides from an Endophytic Fungus *Fusarium avenaceum* W8

**DOI:** 10.3390/molecules29235746

**Published:** 2024-12-05

**Authors:** Zimo Wang, Bo Liu, Yanlei Wang, Yicen Xu, Hai Ma, Yi Sun

**Affiliations:** Institute of Chinese Materia Medica, China Academy of Chinese Medical Sciences, Beijing 100700, China

**Keywords:** endophytic fungus, *Fusarium avenaceum* W8, cyclic depsipeptides, antimicrobial activity, cytotoxic activity

## Abstract

Seven cyclic depsipeptides, including two new cyclic pentadepsipeptides avenamides A (**1**) and B (**2**), were isolated from a plant-derived fungus *Fusarium avenaceum* W8 by using the bioassay-guided fractionation method. The planar structures were elucidated by using comprehensive spectroscopic analyses, including 1D and 2D NMR, as well as MS/MS spectrometry. The absolute configuration of the amino acid and hydroxy acid residues was confirmed by using the advanced Marfey’s method and chiral HPLC analysis, respectively. Compounds **1**–**7** were evaluated for their cytotoxic activities against A549 and NCI-H1944 human lung adenocarcinoma cell lines and their antimicrobial activities against *Staphylococcus aureus* and *Saccharomyces cerevisiae*. As a result, compounds **1**–**4** showed moderate cytotoxicity, with IC_50_ values of 6.52~45.20 µM. Compounds **1** and **3** exhibited significant antimicrobial activities against *S. aureus* and *S. cerevisiae*, with an MIC80 of 11.1~30.0 µg/mL.

## 1. Introduction

Endophytic fungi are potential natural sources of structurally diverse secondary metabolites which possess a variety of biological activities such as antibacterial, anticancer, antiviral, antioxidant, antiparasitic, immunosuppressant, and immunomodulatory activities [1]. The *Fusarium* genus is regarded as a prominent fungal endophyte, for which there have been more than 780 isolated secondary metabolites reported including 185 antibacterial, antifungal, antiviral, and antiparasitic compounds [2,3]. 

*Fusarium* species can produce many types of cyclic depsipeptides, in which at least an amino acid residue is substituted by a hydroxylated carbonxylic acid, structurally ranging from tridepsipeptides to decadepsipeptides [4,5]. Cyclodepsipeptides can be divided into two categories, whose structures are distinguished by whether the arrangement of the amino and ester groups is linked in a regular pattern [6]. Cyclic depsipeptides are considered potential drugs and agrochemicals, and some of them have been developed in clinical trials as antimicrobial and anticancer agents. As one of the typical cyclodepsipeptides with potential biological activities, cyclic pentadepsipeptides have been discovered from the fungal genera of *Fusarium*, *Penicillium*, *Acremonium*, *Alternaria*, and *Hapsidospora*. These compounds are biosynthesized by non-ribosomal peptide synthetases (NRPSs), as well as polyketide or fatty acid synthases. Cyclic pentadepsipeptides have a variety of biological activities including anti-inflammatory, phytotoxic, antifungal, and antiproliferative effects [7]. Some cyclic pentadepsipeptides from *F. solani* have promising anticancer and antibacterial effects. For example, sansalvamide A and its synthetic derivatives showed selective antitumor activity against drug-resistant colon cancer cell lines [8]. In addition, N-methylsansalvamide could exhibit its antitumor effect in HCT116 cells by regulating proliferation, apoptosis, and metastasis in vitro and in vivo [9]. The marine fungus-derived cyclic pentapeptides, aspertides D and E, have potent antibacterial activities against aquatic–pathogenic bacteria [10].

In order to obtain more bioactive lead compounds from endophytic fungi, we chose an endophyte, *avenaceum* W8 (Figure S1), which was derived from the leaves of *Rauvolfia verticillata* Lour by using a bioassay-guided method. This *Fusarium* strain could produce a series of cyclic peptides with antitumor and antimicrobial activities. Herein, we describe the molecular networking analysis of the fungal secondary metabolite extract, the isolation and identification of the new cyclic pentadepsipeptides, and the biological activities of all isolated compounds.

## 2. Results and Discussion

### 2.1. Bioactivity-Guided Isolation and Molecular Networking Analysis

**Figure 1:**
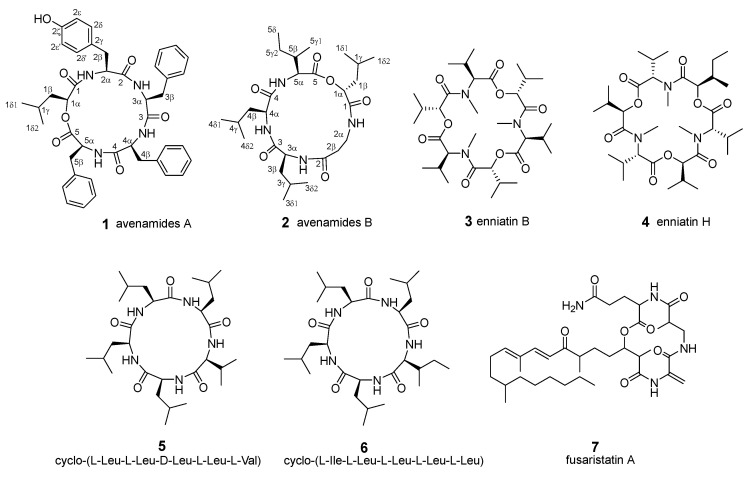
Chemical structures of compounds **1**–**7**.

During our ongoing investigation on the antimicrobial and antitumor lead compounds from endophytic fungi, we isolated seven cyclic peptides from *Fusarium avenaceum* W8 living in the host plant *Rauvolfia verticillata* Lour. As the ethyl acetate (EtOAc) extract of this strain exhibited moderate cytotoxic activity against the A549 human lung adenocarcinoma cell line (IC_50_ value: 36.8 µg/mL), it was fractionated and purified by using a bioassay-guided method (Figure S2). The extract was subsequently subjected to octadecyl-bonded silica (ODS) flash column chromatography using a MeOH-H_2_O solvent system. As a result, the 80% MeOH-H_2_O fraction displayed stronger cytotoxicity than the other fractions, with an IC_50_ value of 27.5 µg/mL. Hence, the 80% MeOH-H_2_O fraction was further subjected to three bioactive subfractions (B-F~B-G). These subfractions were subsequently isolated by using various chromatographic methods to yield two new cyclodepsipeptides, avenamides A (**1**) and B (**2**), together with five known analogues (**3**–**7**), including enniatin B (**3**) [11], enniatin H (**4**) [12], cyclo-(L-Leu-L-Leu-D-Leu-L-Leu-L-Val) (**5**) [13], cyclo-(L-Ile-L-Leu-L-Leu-L-Leu-L-Leu) (**6**) [14], and fusaristatin A (**7**) [15] (Figure 1 and Figure S2). 

**Figure 2:**
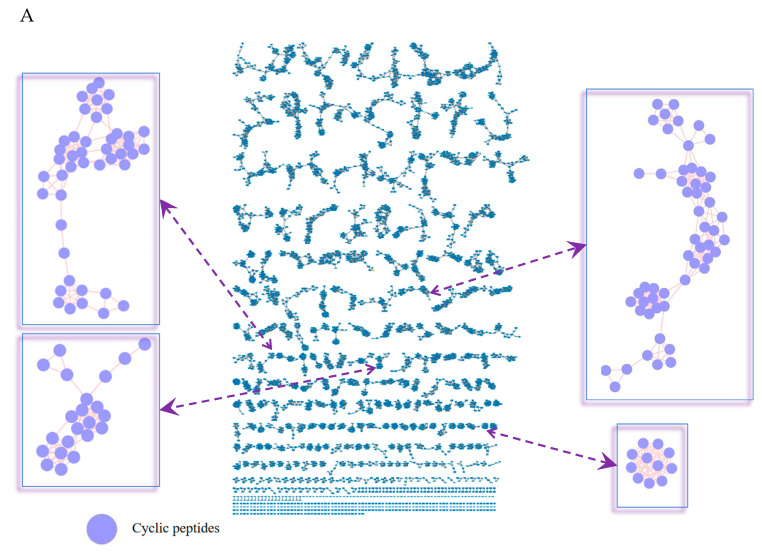

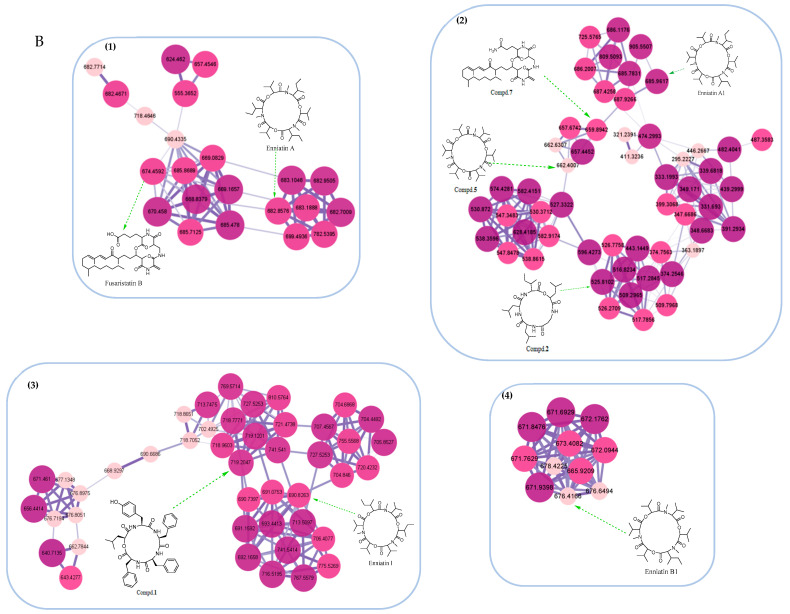
Molecular networking analysis of the EtOAc extract from *F. avenaceum*. (**A**) The molecular networking nodes were matched with the GNPS library, and the clusters in purple were structurally associated with the cyclic peptides. (**B**) The nodes of four clusters (1)–(4) were related to the cyclic depsipeptides of enniatins and avenamides. Enniatin A and fusastatin B were in cluster 1. Compounds **2**, **5**, **7**, and enniatin A1 were in cluster 2. Compound **1** and enniatin I were in cluster 3. Enniatin B1 was in cluster 4.

The EtOAc-soluble extract of *F. avenaceum* W8 was analyzed by UPLC-Q/TOF-MS^E^ in order to prioritize the isolation of cyclic peptides. The required MS^E^ data were processed following the featured-based molecular networking workflow on the GNPS web platform (https://gnps.ucsd.edu [accessed on 28 October 2024]) (Figure 2). The MS/MS library annotated three types of secondary metabolites, including the nodes of cyclic peptides (Figure 2A), alkaloids, and aromatics. Among the clusters, four clusters were expected to be annotated as cyclic depsipeptides, and they predicted the structure of each node as cyclic tetra-, penta-, and hexa- depsipeptide, respectively (Figure 2B). In clusters 1 and 3, the nodes with an *m*/*z* (mass-to-charge) of 719.2 and 525.8 were assumed to be the new cyclodepsipeptides of avenamides A (**1**) and B (**2**), respectively. Additionally, the nodes with an *m*/*z* of 685.962, 676.417, 682.417, 690.826, and 674.460 were automatically matched with the cyclic depsipeptides of enniatins A, A1, B1, H, and fusaristatin B, respectively.

### 2.2. Structural Elucidation of Avenamides A (***1***) and B (***2***)

Avenamide A (**1**) was obtained as a white amorphous powder. Its molecular formula was determined to be C_42_H_46_N_4_O_7_, requiring 22 degrees of unsaturation, by high-resolution electrospray ionization mass spectrometry (HR ESI-MS) (Figure S9) and its nuclear magnetic resonance (NMR) spectra included ^1^H and ^13^C NMR, and 2D NMR (such as heteronuclear single-quantum coherence, HSQC; heteronuclear multiple-bond correlation, HMBC; homonuclear correlation spectroscopy, ^1^H-^1^H COSY; and nuclear Overhauser effect spectroscopy, NOESY) (Table 1 and Figures S3–S8). The ^1^H NMR spectra exhibited the presence of four exchangeable amide NH doublets (*δ*_H_ 8.41, 8.29, 8.05, and 7.91), a hydroxyl singlet (*δ*_H_ 9.22), nineteen aromatic protons (δ_H_ 6.63~7.32) including four coupling aromatic protons of the AA’BB’ system (δ_H_ 6.93, d, *J* = 8.0 Hz, 2H and 6.63, d, *J* = 8.0 Hz, 2H), four α-amino hydrogens (δ_H_ 4.61, 4.39, 4.27, and 4.00), a proton of the oxygenated methine (δ_H_ 4.85), and two doublet peaks (δ_H_ 0.83 and 0.79). The ^13^C NMR data revealed the presence of fourty-two carbon signals, including five amide or ester carbons, twenty-four aromatic carbons, six methines including four α-amino methines and one oxygenated methine, five aliphatic methylenes, and two methyls. On the basis of the above NMR data, **1** was suggested to be a cyclic pentadepsipeptide.

**Figure 3:**
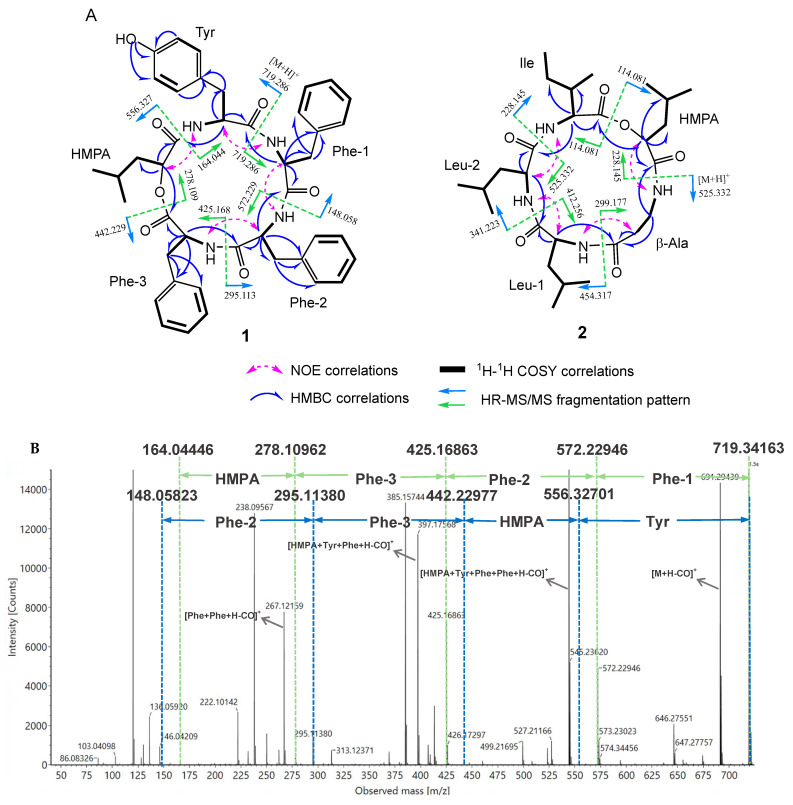

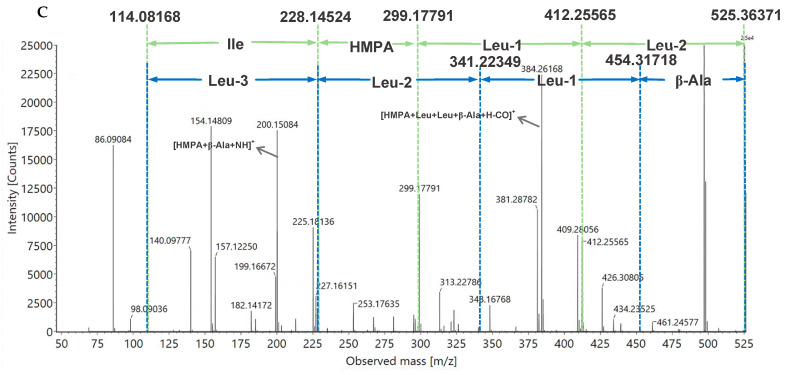
(**A**) Key 2D NMR correlations and MS/MS fragmentation pattern of compounds **1** and **2**. (**B**) MS/MS spectrum of **1**. (**C**) MS/MS spectrum of **2**.

Comprehensive analysis of the 2D NMR data indicated the presence of five distinct fragments to be constructed (Figure 3A), including three Phe residues, one Tyr residue, and one residue of 2-hydroxy-4-methylpentanoic acid (HMPA). The ^1^H-^1^H COSY cross-peaks of H-1α/H-1β/H-1γ/H-1δ1/H-1δ2 and the HMBC correlations from H-1α to C-1, C-1β, and C-1γ suggested the presence of the HMPA moiety. The HMBC correlations from H-2α to C-2β, from H-2β and H-2δ’ to C-2γ, and from H-2ε and OH-2ζ to C-2ζ indicated the presence of the Tyr residue. The three Phe residues were also elucidated by the ^1^H-^1^H COSY, HMBC, and NOESY spectra (Figure 3A). Additionally, the MS/MS fragment of 278.109 [M+H−Phe1−Phe2−Phe3]^+^ also confirmed the continuous substitution of the Phe residues (Figure 3B). The sequence of the aliphatic acid and the amino acid residues was elucidated by the determination of the HMBC and NOESY spectra. The NOESY cross-peaks of H-1α/NH (Tyr), H-2α/NH (Phe1), H-3α/NH (Phe2), and H-4α/NH (Phe3), as well as the HMBC correlation from H-1α to C-5, confirmed the sequence of the amino acids and the hydroxy acid as HMPA-Tyr-Phe1-Phe2-Phe3. Furthermore, the connection was also supported by the analysis of HR-ESI-MS/MS fragmentation (Figure 3B). The HR-ESI-MS/MS experiment determined the major ions by the clockwise direction at an *m*/*z* of 572.229 [M+H−Phe1]^+^, 425.168 [M+H−Phe1−Phe2]^+^, 278.109 [M+H−Phe1−Phe2−Phe3]^+^, and 164.044 [M+H−Phe1−Phe2−Phe3−HMPA]^+^, as well as the fragment ion by the anticlockwise direction at an *m*/*z* of 556.327 [M+H−Tyr]^+^, 442.229 [M+H−HMPA]^+^, 295.113 [M+H−HMPA−Phe3]^+^, and 148.058 [M+H−HMPA−Phe3−Phe2]^+^, indicating the sequence of the amino acids and the hydroxy acid. 

The absolute configuration of each amino acid residue of **1** was determined by using the advanced Marfey’s method. LC-MS analysis of the reaction product confirmed that **1** contained three L-Phes and an L-Tyr after acid hydrolysis and treatment with 1-fluoro-2,4-dinitrophenyl-5-L-alanine amide (FDAA) (Figure S11). The configuration of the hydroxy acid (HMPA) unit was determined as *S* by comparing the hydroxylate of **1** with the standard acids of *R-* and *S*-leucic acids (Figure S10). Thus, compound **1** was named as avenamide A.

The molecular formula of **2** was determined to be C_27_H_48_N_4_O_6_ by HR ESI-MS (Figure S19), requiring six degrees of unsaturation. The ^1^H NMR data (Figure S12) in DMSO-*d6* showed four amide NH protons (δ_H_ 8.46, 8.32, 8.12, and 7.48), five α-amino hydrogens (δ_H_ 4.29, 4.23, 4.03, 3.49, and 3.01), seven doublet methyls (δ_H_ 0.82~0.91), and one triplet methyl (δ_H_ 0.78). The ^13^C NMR and HSQC spectra (Table 1 and Figures S13 and S14) exhibited five amide or ester carbonyls (δ_C_ 172.91, 171.25, 171.09, 169.25, 168.92), eight methines, six methylenes, and eight methyls, which suggested it to be a cyclic pentadepsipeptide.

Further determination of the 2D NMR spectra (Figures S14–S18) of **2** confirmed that it was composed of four amino acid residues including a β-Ala, two Leus, and an Ile, as well as a moiety of 2-hydroxy-4-methylpetanoic acid (HMPA) (Figure 2). The HMPA residue was elucidated by the ^1^H-^1^H COSY cross-peaks of H1α/H-1β/H-1γ/H-1δ1/H-1δ2 and the HMBC correlations from H-1α to C-1 and C-1β. The four amino acid fragments were delineated by the ^1^H-^1^H COSY cross-peaks of H2α/H2β, H3α/H-3β/H-3γ/H-3δ1/H-3δ2, H4α/H-4β/H-4γ/H-4δ1/H-4δ2, and H5α/H-5β/H-5γ1/H-5γ2/H-5δ, as well as the HMBC correlations between the α-hydrogens and their corresponding carbonyls. The HMBC correlations from each α-hydrogen to each carbonyl of the adjacent amino acid and from H-1α to C-5 revealed the linkage sequence of each residue. Further NOESY cross-peaks of NH (β-Ala)/H-1α (Leu-3), NH (Leu-1)/H-2β (β-Ala), NH (Leu-2)/H-1α (Leu-1), and NH (Ile)/H-1α (Leu-2) indicated the subsequent connections of β-Ala−Leu1−Leu2−Ile. 

Furthermore, the sequence of the amino acids and hydroxy acid in **2** was also supported by HR-ESI-MS/MS analysis (Figure 3C), which showed the MS/MS fragment ions at an *m*/*z* of 412.256 [M+H−Leu2]^+^, 299.177 [M+H−Leu2−Leu1]^+^, 228.145 [M+H−Leu2−Leu1−β-Ala]^+^, and 114.081 [M+H−Leu2−Leu1−β-Ala−HMPA]^+^, further identifying the structure of **2**. 

The absolute configuration of the amino acids and hydroxy acid of **2** was also determined by using the Marfey’s method and chiral HPLC analysis, the same as for **1**, which indicated the L-configuration for each amino acid and *R*-leucic acid for the HMPA residue (Figures S20 and S21). 

Compounds **1**–**7** were evaluated for their cytotoxic activities against human lung adenocarcinoma cell lines A549 and NCI-H1944 using the MTT method (Table 2) [5]. Compounds **3** and **4** showed relatively potent cytotoxic activities against A549 cell lines, with IC_50_ values of 13.69 and 6.52 µM, respectively. Compounds **1**–**2** and **7** displayed moderate cytotoxic activities against the two tumor cell lines, whereas **5** and **6** did not possess cytotoxicity, with IC_50_ values less than 50 µM. As shown in Table 2, this indicated that the cyclic pentadepsipeptides (**1**–**4** and **6**) exhibited stronger cytotoxicity against A549 and NCI-H1944 cell lines compared to the other cyclic pentapeptides.

**Figure 4:**
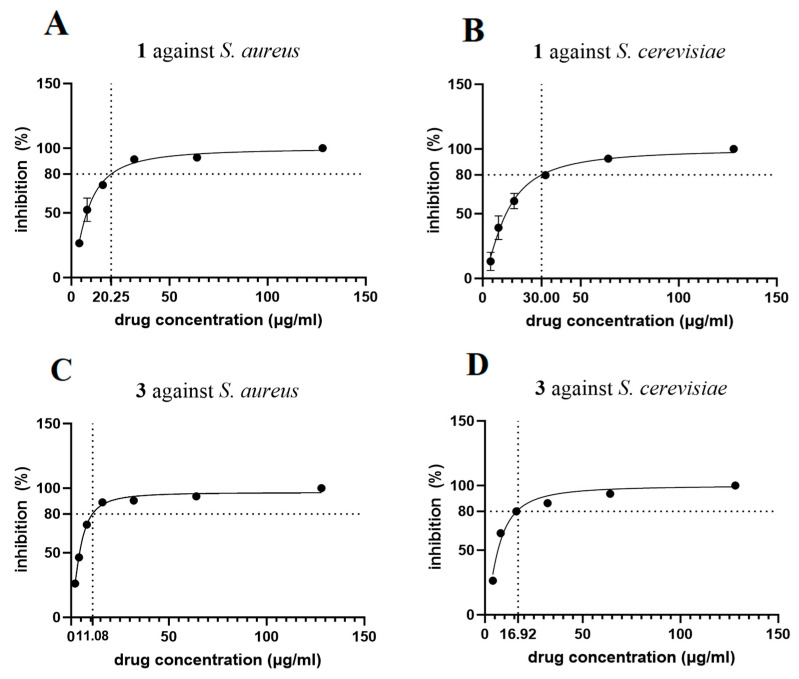
Growth inhibition (%) of compounds **1** (up) and **3** (down) against *S. aureus* (**A**,**C**) and *S. cerevisiae* (**B**,**D**). MIC80 values of **1** and **3** against *S. aureus* were 20.25 and 11.08 μg/mL, respectively; and MIC80 values of **1** and **3** against *S. cerevisiae* were 30.00 and 16.92 μg/mL, respectively.

**Figure 5:**
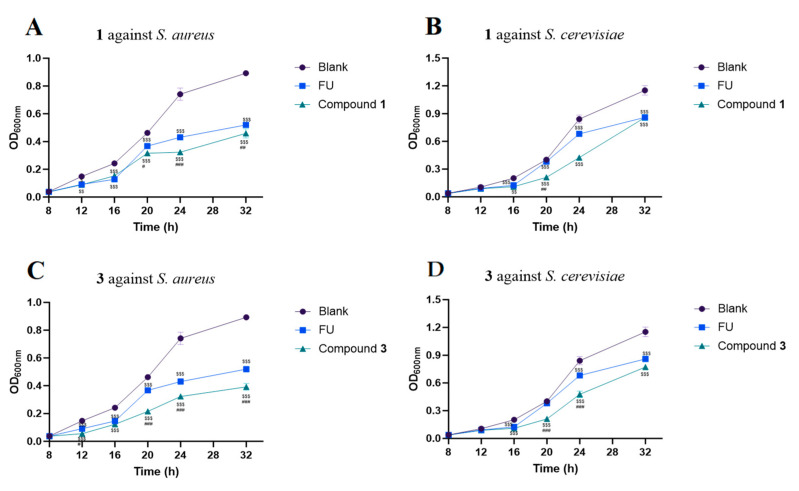
The time–OD600 curves of compounds **1** (up) and **3** (down) for growth inhibition against S. aureus (**A**,**C**) and S. cerevisiae (**B**,**D**). “FU” is the abbreviation of furazolidone, which is used as a positive control drug. ^$^ *p* < 0.05, ^$$^ *p* < 0.01, ^$$$^ *p* < 0.001 vs. Blank; ^#^ *p* < 0.05, ^##^ *p* < 0.01, ^###^ *p* < 0.001 vs. FU. Data were shown as mean ± S.D. (n = 3).

As some of the cyclic depsipeptides possesses antimicrobial acidity, compounds **1**–**7** were also evaluated for their antimicrobial activities against *Staphylococcus aureus* and *Saccharomyces cerevisiae* (Figure 4). As a result, compound **3** exhibited the most significant antimicrobial activity against *S. aureus* and *S. cerevisiae*, with MIC80 values of 11.08 and 16.92 μg/mL, respectively. In addition, **1** could also inhibit the growth of both strains, with MIC80 values of 20.25 and 30.00 μg/mL, respectively. The growth time curves of *S. aureus* and *S. cerevisiae* were measured with **1** and **3** at their MIC80 concentrations over 32 h (Figure 5). Compounds **1** and **3** exhibited antimicrobial activity against both strains during the measuring time. Both **2** and **4** containing an isoleucine residue did not display strong antimicrobial activity against the tested pathogenic strains, suggesting that the isoleucine residue might have no contribution to the inhibitory effect.

## 3. Materials and Methods

### 3.1. General Experimental Procedures

NMR spectra were recorded on a Bruker ARX-600 spectrometer (600 MHz, Bruker Co., Ltd., Karlsruhe, Germany) in DMSO-*d*_6_. All compounds were determined by 1D and 2D NMR, which included ^1^H and ^13^C NMR, as well as HSQC, HMBC, ^1^H-^1^H COSY, TOCSY, and NOESY spectra. UPLC-MS, HRESI-MS, and MS/MS data were obtained on a Waters ACQUITY UPLC H-Class/Vion QTOF mass spectrometer (Waters Micromass, Manchester, UK). Semipreparative HPLC separation was performed on an Agilent 1260 pump (Agilent Technologies Co., Ltd., Palo Alto, CA, USA) coupled with an Agilent semipreparative Cosmosil C_18_ column (10 mm × 250 mm, 5 μm, Co., Ltd., Nacalai Tesque, Kyoto, Japan). RP C_18_ silica gel (ODS-A, S-50 μm, YMC, Co., Ltd., Kyoto, Japan) and Sephadex LH-20 (Pharmacia Fine Chemical Co., Ltd., Uppsala, Sweden) were used for column chromatography (CC). Human carcinoma cell lines A549 and NCI-H1944 were purchased from the Chinese National Infrastructure of Cell Line Resource (NICR). *S. aureus* and *S. cerevisiae* were purchased from the China General Microbiological Culture Collection and Management Center. Adriamycin (ADM) was purchased from Shanghai Yuanye Biotechnology Co., Ltd. (Shanghai, China). PDA and LB media were purchased from Becton, Dickinson Company (Franklin Lakes, NJ, USA).

### 3.2. Fungal Material

The strain *Fusarium avenaceum* was isolated from the fresh leaf of *Rauvolfia verticillata* Lour. Baill. collected in Xishuangbanna, China, in 2019 (Figure S1). Fungal characterization was performed on the basis of the morphological and molecular characteristics. The analysis of the internal transcribed spacer (ITS1-5.8S-ITS2) rDNA gene revealed that the ITS sequence of the strain *Fusarium avenaceum* showed the highest homology. A BLAST search result showed that the sequence was most similar (99.44%) to the sequence of *Fusarium avenaceum* (compared to MW620135.1).

### 3.3. Fermentation and Isolation

The fungus was inoculated on PDA plates and cultured at 27 °C for 7 days. PDA agar blocks were inoculated in 150 bottles of rice medium and cultured in a stationary status at 27 °C for 16 days. After growing the strains for the indicated number of days, the culture was quenched with large amounts of ethyl acetate, sonicated for 20 min, and extracted twice in duplicate. The solvent was recovered under reduced pressure to obtain the crude ethyl acetate extract, fully dissolved with a little methanol and then extracted with an equal volume of petroleum ether to remove the excess oil produced by rice cultivation. This enabled the recovery of the methanol layer and the petroleum ether layer.

The crude extract (1.6 g) of *F. avenaceum* W8 was fractionated by ODS flash column chromatography eluted with 40%, 60%, 80%, and 100% (*v*/*v*) MeOH in H_2_O, yielding 40% (428.7 mg), 60% (107.1 mg), 80% (510 mg), and 100% (508.7 mg) MeOH-H_2_O fractions. The fraction eluted with 80% MeOH was isolated by using the silica gel column to give four subfractions (A−D), and then fraction 80B was further separated by Sephadex LH-20 (CHCl_2_:MeOH = 1:1) column chromatography to give five subfractions (E–I). 

Because some cyclic peptide compounds have weak UV absorption, the differential refractive detector was used to determine the semipreparation conditions. Thus, subfraction 80B-F was purified by semipreparative HPLC (Kromasil Eternity XT-5-C_18_ column, Akzo Nobel Co., Ltd., Göteborg, Sweden, 250 mm × 10 mm i.d., 5 μm, 3mL·min^−1^) with 65% ACN in H_2_O with 0.2% AcOH to afford compound **3** (*t*_R_ = 28.5 min, 1.5 mg). Subfraction 80B-G was purified by semipreparative HPLC (Kromasil Eternity XT-5-C_18_ column, 250 mm × 10 mm i.d., 5 μm, 3mL·min^−1^) with 65% ACN in H_2_O with 0.2% AcOH to afford compounds **2** (*t*_R_ = 20.5 min, 1.5 mg), **6** (*t*_R_ = 28 min, 2.1 mg), and **7** (*t*_R_ = 41.3 min, 3.3 mg). Subfraction 80B-H was purified by semipreparative HPLC (Cosmosil MSII C_18_ column, NacalaiTesque Co., Ltd., Kyoto, Japan, 250 mm × 10 mm i.d., 5 μm, 3mL·min^−1^) with 55% ACN in H_2_O with 0.2% AcOH to afford compounds **1** (*t*_R_ = 20.5 min, 1.1 mg), **4** (*t*_R_ = 25.7 min, 1.1 mg), and **5** (*t*_R_ = 45.2 min, 2.4 mg).

Compound **1**. White powder; ${\left[\alpha \right]}_{D}^{25}$ −0.031 (*c* 0.01, MeOH). For ^1^H and ^13^C NMR data (DMSO-*d*_6_), see Table 1 and Figures S3 and S4. HRMS (ESI-TOF) *m*/*z*: [M+H]^+^ Calcd for C_42_H_47_N_4_O_7_ 719.34163. Found 719.34393, Δ2.3 ppm.

Compound **2**. White powder; ${\left[\alpha \right]}_{D}^{25}$ −0.023 (*c* 0.01, MeOH). For ^1^H and ^13^C NMR data (DMSO-*d*_6_), see Table 1 and Figures S12 and S13. HRMS (ESI-TOF) *m*/*z*: [M+H]^+^ Calcd for C_27_H_48_N_4_O_6_ 525.36371. Found 525.36466, Δ1.8 ppm.

### 3.4. Advanced Marfey’s Analysis and Chiral HPLC Analysis

Marfey’s method was used to determine the absolute configurations of hydrolytic amino acid residues in natural products of linear and cyclic peptides. The procedure of the reaction was as follows. Compound **1** or **2** (0.5 mg) in a 3 mL screwed bottle was added with 0.5 mL of 6 N HCl solution. The vial was hydrolyzed in a 110 °C oil bath for 16 h. Then, the hydrolysis products were added with 100 μL of 1% FDAA acetone solution and 50 μL of 1 N NaHCO_3_. After the mixed solution was successively heated in a 50 °C water bath for 1 h, the reaction was terminated by adding 50 μL of 1 N HCl.

Each amino acid standard (1 mg) was prepared in an EP tube, and then 100 μL of 1% FDAA acetone solution and 50 μL of 1 N NaHCO_3_ were added. The mixture was heated in a 50 °C water bath for 1 h, and then the reaction was terminated by adding 50 μL of 1 N HCl. 

(*R*)-leucic acid and (*S*)-leucic acid standards were analyzed separately using a CHIRALPAKID chiral column, eluting with 5% (*v*/*v*) isopropanol in *n*-hexane containing 0.2%~0.3% formic acid, at a flow rate of 0.8 mL/min in 20min.

### 3.5. MS/MS Data Conversion and Molecular Network Visualization

We used progenesis QI software 2.0 to convert the raw MS/MS data into csv and msp formats. The converted data files were uploaded and processed via “Feature networking” on the GNPS Web platform (http://gnps.ucsd.edu [accessed on 28 October 2024]). We set the index according to the following parameters: precursor mass tolerance: 0.02 Da; MS/MS fragment ion tolerance: 0.02 Da; minimum cosine score: 0.3; minimum matched fragment ions: 7; minimum cluster size: 1; network TopK: 10. Cytoscape v3.9.1 was used to visualize the generated molecular network. The molecular network visualization can be publicly accessed at https://gnps.ucsd.edu/ProteoSAFe/status.jsp?task=12486ebb65354c809f3872e0e701e569 (accessed on 28 October 2024).

### 3.6. UPLC-HR-MS Data Acquisition

UPLC-HR-MS data were obtained by using a C18 Acquity UPLC BEH column (2.1 × 50 mm, 1.7 μm, Waters, Milford, MA, USA). Gradient elution was performed by using 0.2% formic acid aqueous solution (A) and acetonitrile (B) as the mobile phases, and the solvent started from 10% B to 90% B for 15 min. Subsequently, the washing step was performed at 90% B for 2 min. The flow rate was 0.3 mL/min and the injection volume was 1 μL. The mass spectra were recorded in positive ion mode in the range of *m*/*z* 50–1000. The ESI source parameters were applied with the following parameters. capillary voltage: 3 kV; cone voltage: 40 V; source offset: 80 V; desolvation temperature: 450 °C; source temperature: 120 °C; desolvation gas: 800 L/h; cone gas: 50 L/h. The low collision energy was 6 eV. The high collision energy ramp was 20–40 eV.

### 3.7. Cytotoxicity Assay

The cytotoxicity of the compound against A549 and NCI-1944 lines was determined by using 3-(4,5-dimethyl-2-thiazolyl)-2,5-diphenyl-2-H-tetrazolium bromide (MTT). The cells were maintained in DMEM containing 10% (*v*/*v*) fetal bovine serum (FBS) and 0.4% (*v*/*v*) penicillin−streptomycin solution (10,000 units/mL penicillin and 10,000 μg/mL streptomycin, 100X) at 37 °C under 5% CO_2_. After 24 h of adherent cell growth, different concentrations of the compounds, positive control adriamycin (ADM), and blank control (methanol) were added to 96-well plates. After 72 h, 20 μL of MTT solution was added. The absorbance value (detection wavelength is 562 nm and reference wavelength is 630 nm) was measured on the microplate reader, and the IC_50_ value of each compound was calculated.

### 3.8. Antibacterial Activity Assay

Strains of *S. aureus* and *S. cerevisiae* were used for the antimicrobial activity assay, and compounds **1**–**7** were evaluated as described. We resuscitated the bacteria and centrifuge them at 8000 rpm for 5 min at 4 °C; we then washed the precipitate twice with PBS. We diluted it with Luria-Bertani (LB) broth medium to the concentration of 5 × 10^5^ CFU/mL. Strains were grown to OD_600_ = 0.3 in LB broth medium and diluted 400-fold. Aliquots (200 μL) of the diluted suspensions were placed into 96-well microtiter plates with isolated compounds at concentrations of 128, 64, 32, 16, 8, and 4 μg/mL and cultured at 37 °C for 24 h. Furazolidone was the positive control and DMSO was used as the negative control. The OD_600_ values were used to test the antimicrobial rate for MIC80. 

The strain was grown to OD_600_ = 0.3 in LB broth medium and diluted 100-fold. Aliquots (200 μL) of the diluted suspensions were placed into 96-well microtiter plates, and compounds **1** and **4** at the concentration of MIC80 were cultured in the plate at 37 °C. The absorbance value was measured at OD_600_ at 8 h, 12 h, 16 h, 20 h, 24 h, and 32 h.

## 4. Conclusions

The reported cyclic pentadepsipeptides containing the leucic acid residue possessed significant cytotoxicity against A549, SK-OV-3, SK-MEL-2, and MES-SA human tumor cell lines [16,17]. Moreover, a cyclic pentadepsipeptide named leualacin was a calcium channel blocker [18]. During our chemical investigation of the endophytic fungus *Fusarium avenaceum* W8, seven cyclic peptides were isolated, including two new leucic acid-containing cyclic pentadepsipeptides, avenamides A (**1**) and B (**2**), together with five known analogues, enniatin B (**3**) and enniatin H (**4**), cyclo-(L-Leu-L-Leu-D-Leu-L-Leu-L-Val) (**5**), cyo-(L-Ile-L-Leu-L-Leu-L-Leu) (**6**), and fusaristatin A (**7**). The new structures were elucidated by comprehensive 1D and 2D NMR analyses and MS/MS fragmentation, and their stereochemical structures were determined by using comprehensive spectroscopic analyses, the advanced Marfey’s method, and chiral HPLC analysis. This discovery broadens the structural diversity of the leucic acid-containing cyclodepsipeptides in nature.

The cytotoxicity and antimicrobial activity of all the cyclic peptides were evaluated. Compounds **1** and **3** displayed significant inhibitory effects against *S. aureus* and *S. cerevisiae* within 32 h. This study also demonstrated that compounds **1**–**4** and **7** exhibited moderate cytotoxicity against two human lung tumor cell lines A549 and NCI-H1944, with IC_50_ values less than 50 µM (Table 1). Our findings suggest that bioactive cyclodepsipeptides have the potential to be developed as antimicrobial and antitumor agents for the treatment of diseases.

## Figures and Tables

**Table 1:** ^1^H-NMR and ^13^C-NMR data of compounds **1** and **2** in DMSO-*d_6_.*

1			2		
No.	δ_C_	δ_H_, mult. (J in Hz)	No.	δ_C_	δ_H_, mult. (J in Hz)
**OLeu**			**OLeu**		
1	168.5	−	1	168.9	-
2	73.9	4.85 (dd, *J* = 8.3, 5.8 Hz, 1H)	2	72.9	5.1 (dd, *J* = 7.3, 4.6 Hz, 1H)
3	40.2	1.58 (dd, *J* = 14.3, 7.5 Hz, 1H); 1.42 (dd, *J* = 14.3, 5.8 Hz, 1H)	3	40.7	1.54–1.65 (m, 2H)
4	23.7	1.30 (m, 1H)	4	24.1	1.62 (m, 1H)
5	22.5	0.83 (d, *J* = 6.5 Hz, 3H)	5	23.4	0.81 (d, *J* = 6.7 Hz, 3H)
6	21.9	0.79 (d, *J* = 6.5 Hz, 3H)	6	23.4	0.86 (d, *J* = 6.8 Hz, 3H)
**Tyr**			**β-Ala**		
1	170.6	−	1	171.1	-
2	54.9	4.39 (dd, *J* = 8.8, 6.6 Hz, 1H)	2	35.4	3.49 (m, 1H); 3.01 (m, 1H)
3	36.3	2.79 (m, 1H); 2.85 (m, 1H)	3	34.9	2.4 (t, *J* = 13.7 Hz, 1H); 2.12 (dt, *J* = 14.9, 3.1 Hz, 1H)
4	127.1	−	NH		7.48 (brs, 1H)
5	129.9	6.93 (d, *J* = 8.0 Hz, 1H)			
6	115.0	6.63 (d, *J* = 8.0 Hz, 1H)			
7	155.9	−			
8	115.0	6.63 (d, *J* = 8.0 Hz, 1H)			
9	129.9	6.93 (d, *J* = 8.0 Hz, 1H)			
NH		7.91 (d, *J* = 9.3 Hz, 1H)			
OH		9.22 (s, 1H)			
**Phe1**			**Leu1**		
1	171.0	-	1	171.2	-
2	57.1	4.00 (m, 1H)	2	51.1	4.23 (m, 1H)
3	36.0	2.85 (m, 1H); 3.02 (m, 1H)	3	40.1	1.57–1.66 (m, 2H)
4	137.5	-	4	24.5	1.50–1.60 (m, 1H)
5	129.0	7.12 (d, *J* = 7.4 Hz, 1H)	5	22.3	0.83 (d, *J* = 6.8 Hz, 3H)
6	128.3	7.28 (m, 1H)	6	23.3	0.91 (d, *J* = 6.8 Hz, 3H)
7	126.5	7.19 (m, 1H)	NH		8.12 (brs, 1H)
8	128.3	7.28 (m, 1H)			
9	129.0	7.12 (d, *J* = 7.4 Hz, 1H)			
NH		8.29 (s, 1H)			
**Phe2**			**Leu2**		
1	170.8	-	1	172.9	-
2	56.2	4.27 (m, 1H)	2	52.0	4.29 (m, 1H)
3	37.1	2.88 (m, 2H)	3	41.1	1.46–1.50 (m, 2H)
4	137.4	-	4	24.3	1.60–1.66 (m, 1H)
5	128.9	6.99 (d, *J* = 7.0 Hz, 1H)	5	21.6	0.89 (d, *J* = 6.6 Hz, 3H)
6	128.3	7.20 (m, 1H)	6	22.0	0.88 (d, *J* = 6.8 Hz, 3H)
7	126.4	7.21 (m, 1H)	NH		8.46 (brs, 1H)
8	128.3	7.20 (m, 1H)			
9	128.9	6.99 (d, *J* = 7.0 Hz, 1H)			
NH		8.05 (s, 1H)			
**Phe3**			**Ile**		
1	168.8	-	1	169.2	-
2	54.2	4.61 (q, *J* = 8.0 Hz, 1H)	2	57.9	4.03 (m, 1H)
3	36.5	3.09 (dd, *J* = 13.8, 9.0 Hz, 1H); 3.18 (dd, *J* = 13.8, 6.5 Hz, 1H)	3	34.3	2.04 (m, 1H)
4	136.8	-	4	24.2	1.03 (dq, *J* = 17.4, 9.9, 8.4 Hz, 1H); 1.44 (m, 1H)
5	129.2	7.26 (m, 1H)	5	15.6	0.86 (d, *J* = 6.7 Hz, 3H)
6	128.4	7.32 (t, *J* = 7.5 Hz, 1H)	6	10.0	0.78 (t, *J* = 7.5 Hz, 3H)
7	126.7	7.22 (m, 1H)	NH		8.32 (brs, 1H)
8	128.4	7.32 (t, *J* = 7.5 Hz, 1H)			
9	129.2	7.26 (m, 1H)			
NH		8.41 (d, *J* = 8.5 Hz, 1H)			

**Table 2:** Cytotoxicity results of **1**–**2** and **5**–**7** against A549 and NCI-H1944 cell lines.

	IC_50_ Values (μM) of 1–4 and 7 ^a^					
Cell lines	**1**	**2**	**3**	**4**	**7**	adriamycin
*A549*	38.60 ± 0.40	45.20 ± 0.42	13.69 ± 0.27	6.52 ± 0.25	26.36 ± 0.32	4.26 ± 0.17
*NCI-H1944*	28.60 ± 0.12	37.66 ± 0.15	23.66 ± 0.37	24.60 ± 0.20	39.50 ± 0.30	4.35 ± 0.27

^a^ Adriamycin and DMSO were used as the positive and negative controls, respectively.

## Data Availability

Bioguided isolation flow chart of the extract of *F. avenaceum* and the NMR data for compounds **1** and **2** are contained within the article and Supplementary Materials.

## Supplementary Materials

The following supporting information can be downloaded at https://www.mdpi.com/article/10.3390/molecules29235746/s1: Figure S1: The strain of *Fusarium avenaceum*; Figure S2: Bioguided isolation flow chart of extract of *F. avenaceum*; Figure S3: ^1^H NMR spectrum of compound **1** at 600 MHz in DMSO*-d_6_*; Figure S4: ^13^C NMR spectrum of compound **1** at 150 MHz in DMSO*-d_6_*; Figure S5: HSQC spectrum of compound **1** at 600 MHz in DMSO*-d_6_*; Figure S6: HMBC spectrum of compound **1** at 600 MHz in DMSO*-d_6_*; Figure S7: ^1^H-^1^H COSY spectrum of compound **1** at 600 MHz in DMSO*-d_6_*; Figure S8: NOESY spectrum of compound **1** at 600 MHz in DMSO*-d_6_*; Figure S9: HRESI-MS spectrum of compound **1**; Figure S10: The HPLC chart of the hydrolysate of compound **1** and the standards (*R*)/(*S*)-leucic acid; Figure S11: LC-MS analysis of the Marfey’s products of compound **1** and the amino acid standards; Figure S12: ^1^H NMR spectrum of compound **2** at 600 MHz in DMSO*-d_6_*; Figure S13: ^13^C NMR spectrum of compound 2 at 150 MHz in DMSO*-d_6_*; Figure S14: HSQC spectrum of compound **2** at 600 MHz in DMSO*-d_6_*; Figure S15: HMBC spectrum of compound **2** at 600 MHz in DMSO*-d_6_*; Figure S16: ^1^H-^1^H COSY spectrum of compound **2** at 600 MHz in DMSO*-d_6_*; Figure S17: TOCSY spectrum of compound **2** at 600 MHz in DMSO*-d_6_*; Figure S18: NOESY spectrum of compound **2** at 600 MHz in DMSO*-d_6_*; Figure S19: HRESI-MS spectrum of compound **2**; Figure S20: The HPLC chart of the hydrolysate of compound **2** and the standards (*R*)/(*S*)-leucic acid; Figure S21: LC-MS analysis of the Marfey’s products of compound **2** and the amino acid standards.
