# Supplementary material for: Cytotoxic and Antibacterial Cyclodepsipeptides from an Endophytic Fungus Fusarium avenaceum W8
Source: Molecules. 2024 Dec 5;29(23):5746. doi: 10.3390/molecules29235746 (PMC11643578; doi:10.3390/molecules29235746)

Cytotoxic and Antibacterial Cyclodepsipeptides from an Endophytic Fungus *Fusarium avenaceum* W8

Zimo Wang †, Bo Liu †, Yanlei Wang, Yicen Xu, Hai Ma * and Yi Sun *

Institute of Chinese Materia Medica, China Academy of Chinese Medical Sciences, Beijing 100700, China; wangzimo0913@163.com (Z.W.); bliu@icmm.ac.cn (B.L.); w15739539626@163.com (Y.W.); 15391696684@163.com (Y.X.)

* Correspondence: hma@icmm.ac.cn (H.M.); ysun@icmm.ac.cn (Y.S.)

† The authors contribute equally to this work.

Contents

Figure S1. The strain of *Fusarium avenaceum*..3

Figure S2. Bioguided isolation flow chart of the extract of *F. avenaceum*..3

Figure S3. ^{1}H NMR spectrum of compound 1 at 600 MHz in DMSO-*d_6*..4

Figure S4. ^{13}C NMR spectrum of compound 1 at 150 MHz in DMSO-*d_6*..5

Figure S5. HSQC spectrum of compound 1 at 600 MHz in DMSO-*d_6*...6

Figure S6. HMBC spectrum of compound 1 at 600 MHz in DMSO-*d_6*..7

Figure S7. ^{1}H-^{1}H COSY spectrum of compound 1 at 600 MHz in DMSO-*d_6*...8

Figure S8. NOESY spectrum of compound 1 at 600 MHz in DMSO-*d_6*..9

Figure S9. HRESI-MS spectrum of Compound 1...10

Figure S10. HPLC chart of the hydrolysate of compound 1 and the standards(*R*)/(*S*)-Leucic acid.................11

Figure S11. LC-MS analysis of the Marfey′ s products of compound 1 and amino acid standards.................11

Figure S12. ^{1}H NMR spectrum of compound 2 at 600 MHz in DMSO-*d_6*...12

Figure S13. ^{13}C NMR spectrum of compound 2 at 150 MHz in DMSO-*d_6*..13

Figure S14. HSQC spectrum of compound 2 at 600 MHz in DMSO-*d_6*..14

Figure S15. HMBC spectrum of compound 2 at 600 MHz in DMSO-*d_6*...15

Figure S16. ^{1}H-^{1}H COSY spectrum of compound 2 at 600 MHz in DMSO-*d_6*..16

Figure S17. TOCSY spectrum of compound 2 at 600 MHz in DMSO-*d_6*..17

Figure S18. NOESY spectrum of compound 2 at 600 MHz in DMSO-*d_6*..18

Figure S19. HRESI-MS spectrum of Compound 2...19

Figure S20. HPLC chart of the hydrolysate of compound 2 and the standards(*R*)/(*S*)-Leucic acid.................19

Figure S21. LC-MS analysis of the Marfey′ s products of compound 2 and amino acid standards..............20

Supporting Information

Figure S1. The strain of *Fusarium avenaceum* and its ITS sequence

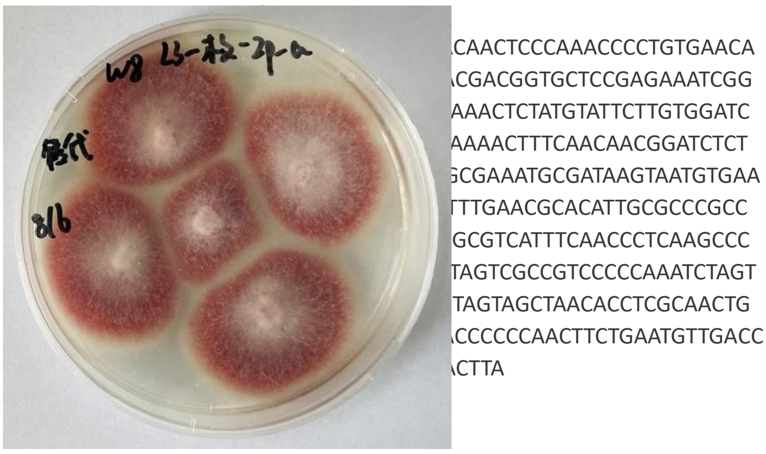

CAACTCCCAAACCCCTGTGAACA
CGACGGTGCTCCGAGAAATCGG
AAACTCTATGTATTCTTGTGGATC
AAAACTTTCAACAACGGATCTCT
CGAAATGCGATAAGTAATGTGAA
TTGAACGCACATTGCGCCCGCC
GCGTCATTTCAACCCTCAAGCCC
TAGTCGCCGTCCCCCAAATCTAGT
TAGTAGCTAACACCTCGCAACTG
CCCCCCAACTTCTGAATGTTGACC
CTTA

Figure S2. Bio-guided isolation flow chart of the extract of *F. avenaceum*

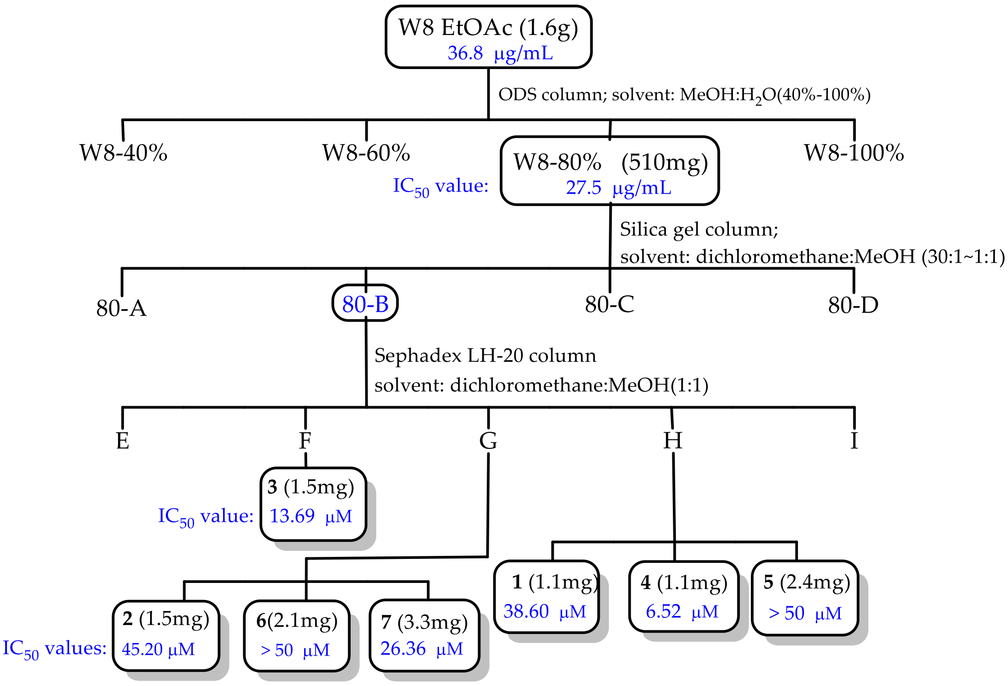

Figure S3. ¹H NMR spectrum of compound 1 at 600 MHz in DMSO-d_6

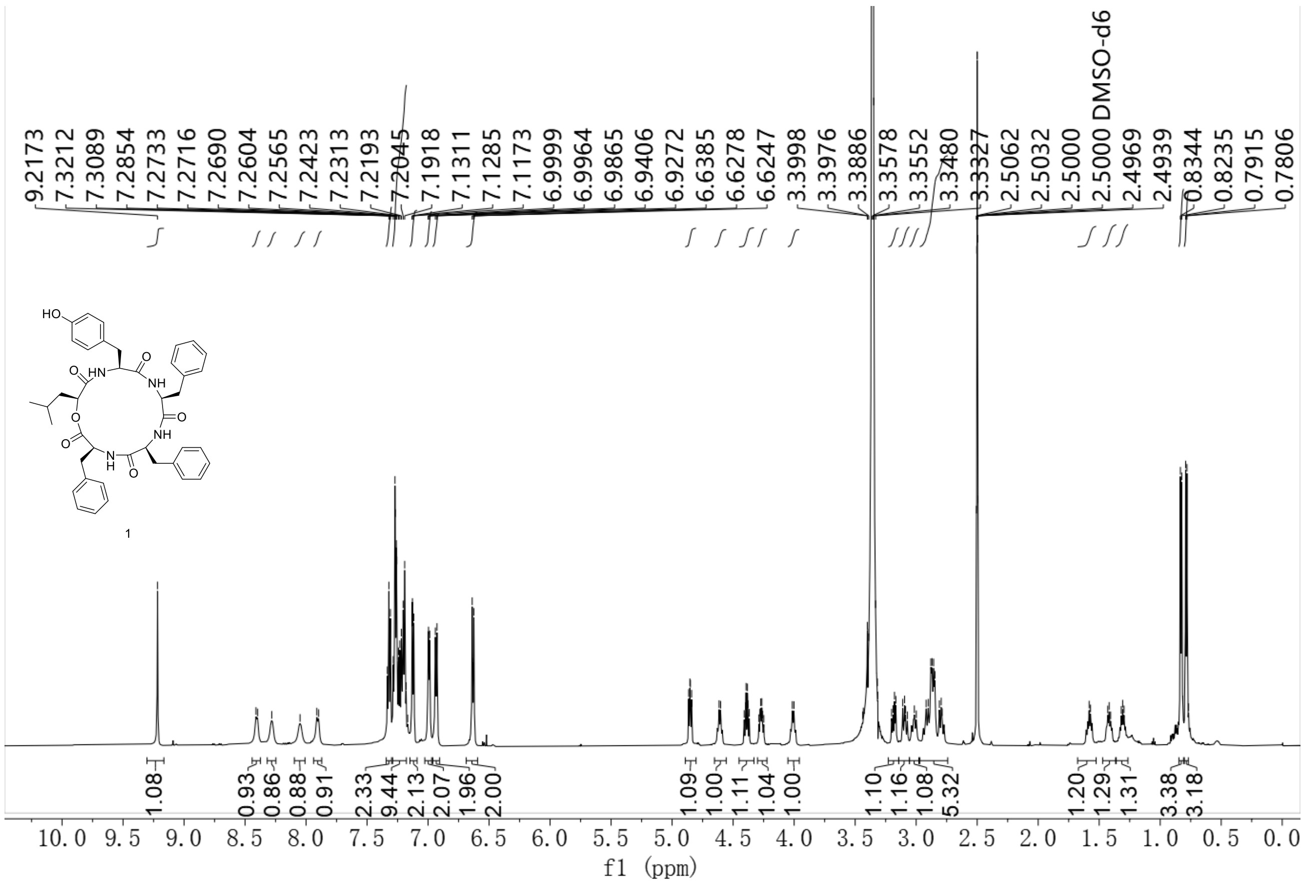

Figure S4. ^{13}C NMR spectrum of compound 1 at 150 MHz in DMSO-d_6

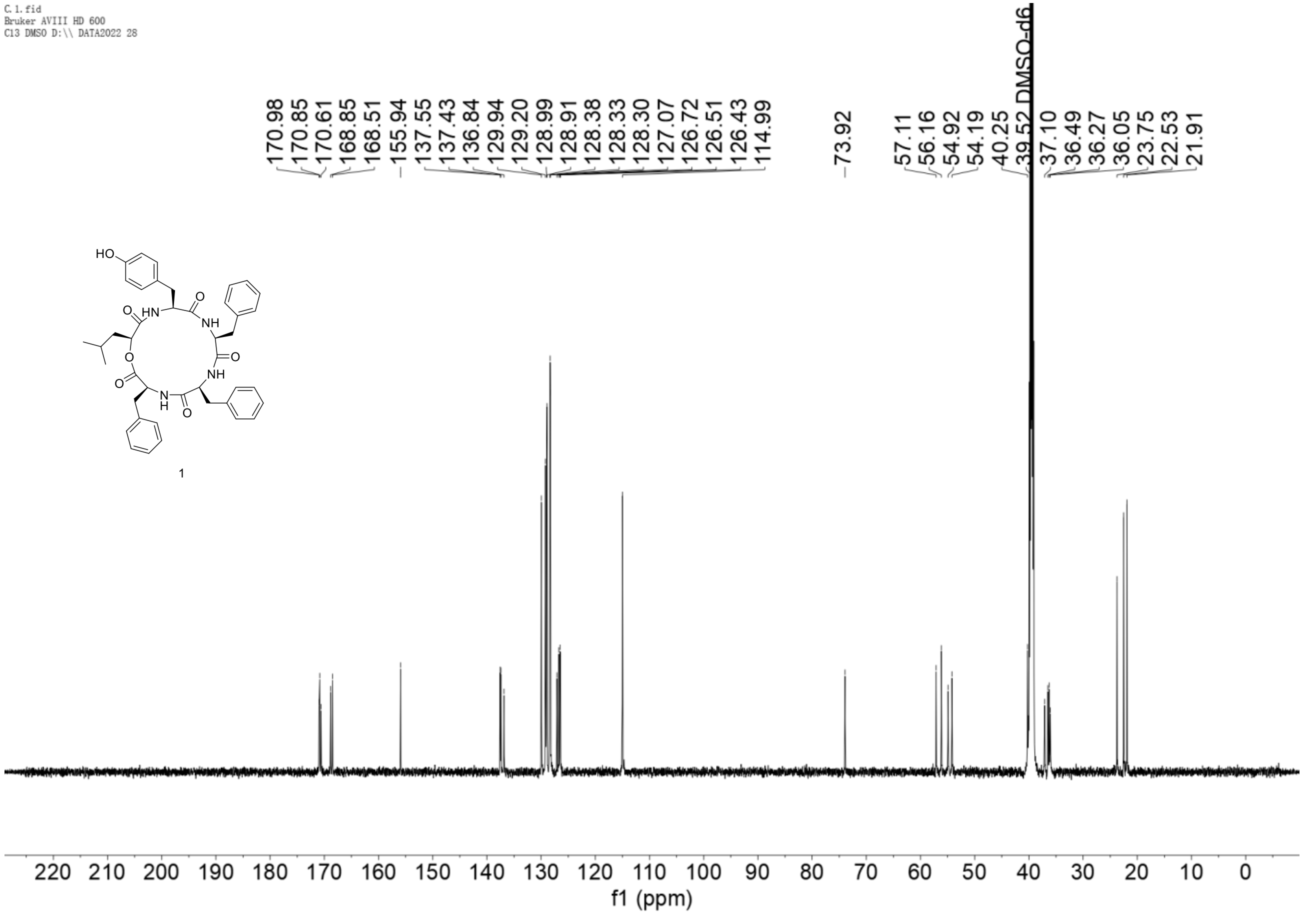

1

Figure S5. HSQC spectrum of compound 1 at 600 MHz in DMSO-d_6

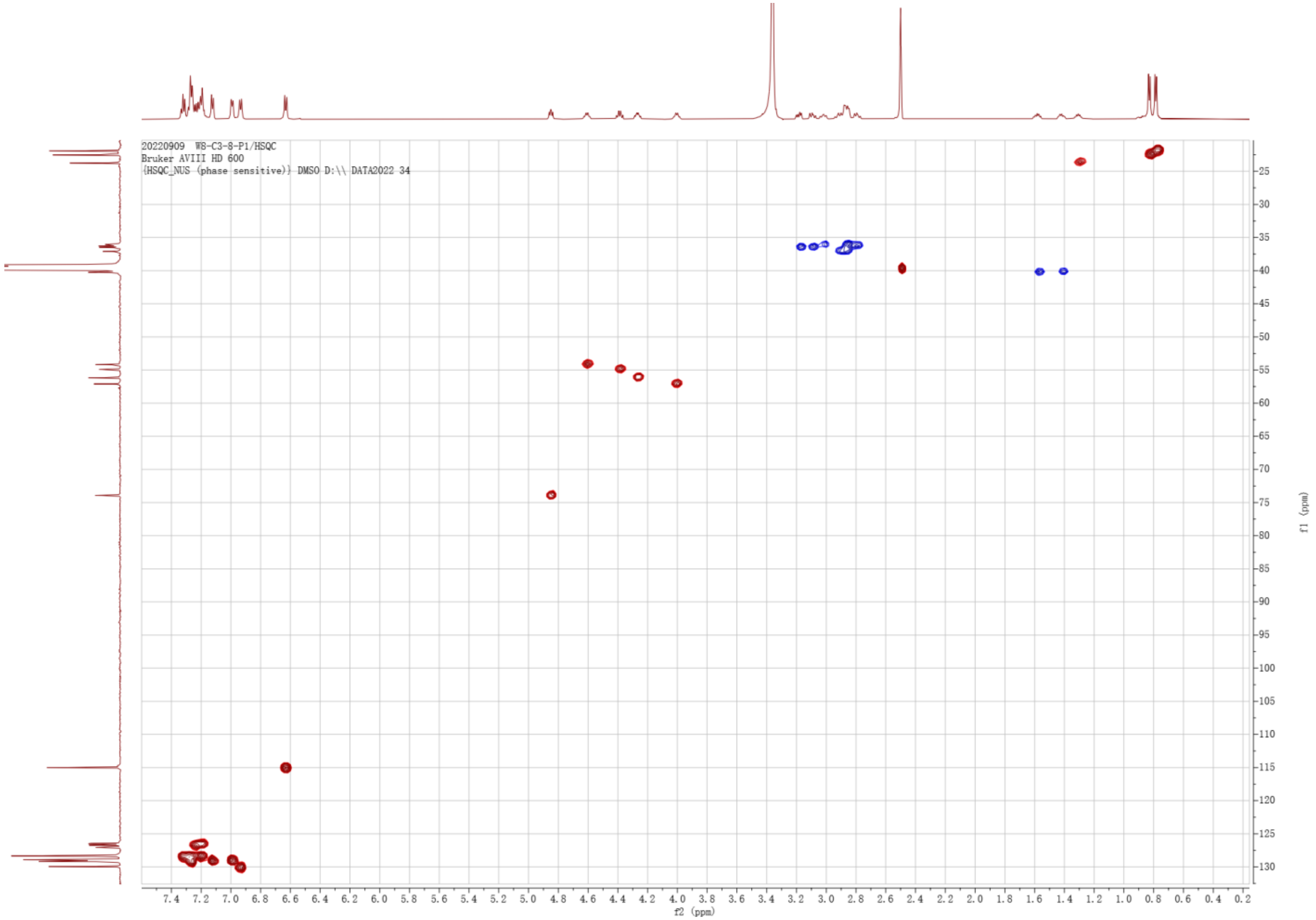

Figure S6. HMBC spectrum of compound 1 at 600 MHz in DMSO-d_6

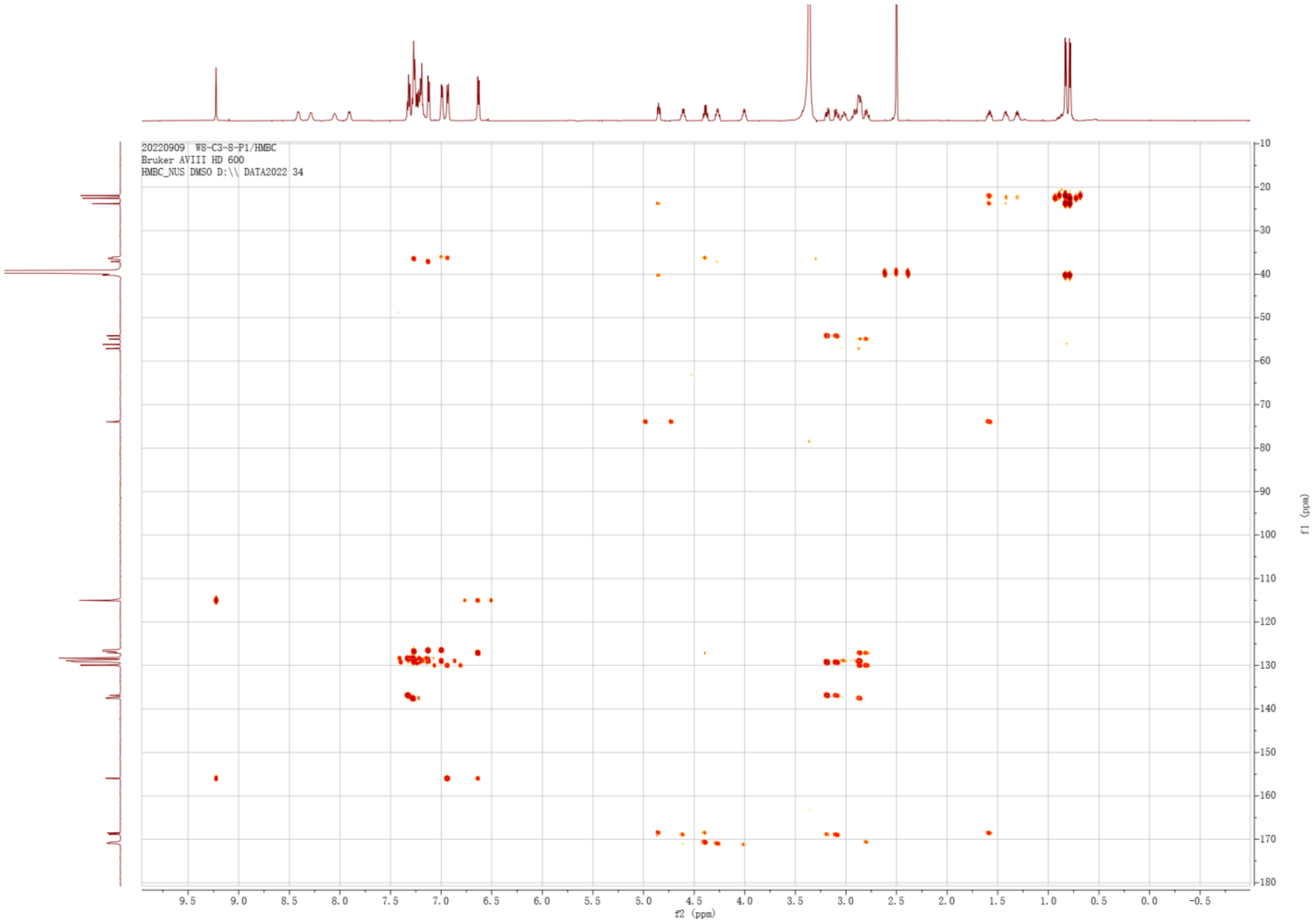

Figure S7. ^{1}H-^{1}H COSY spectrum of compound 1 at 600 MHz in DMSO-d_6

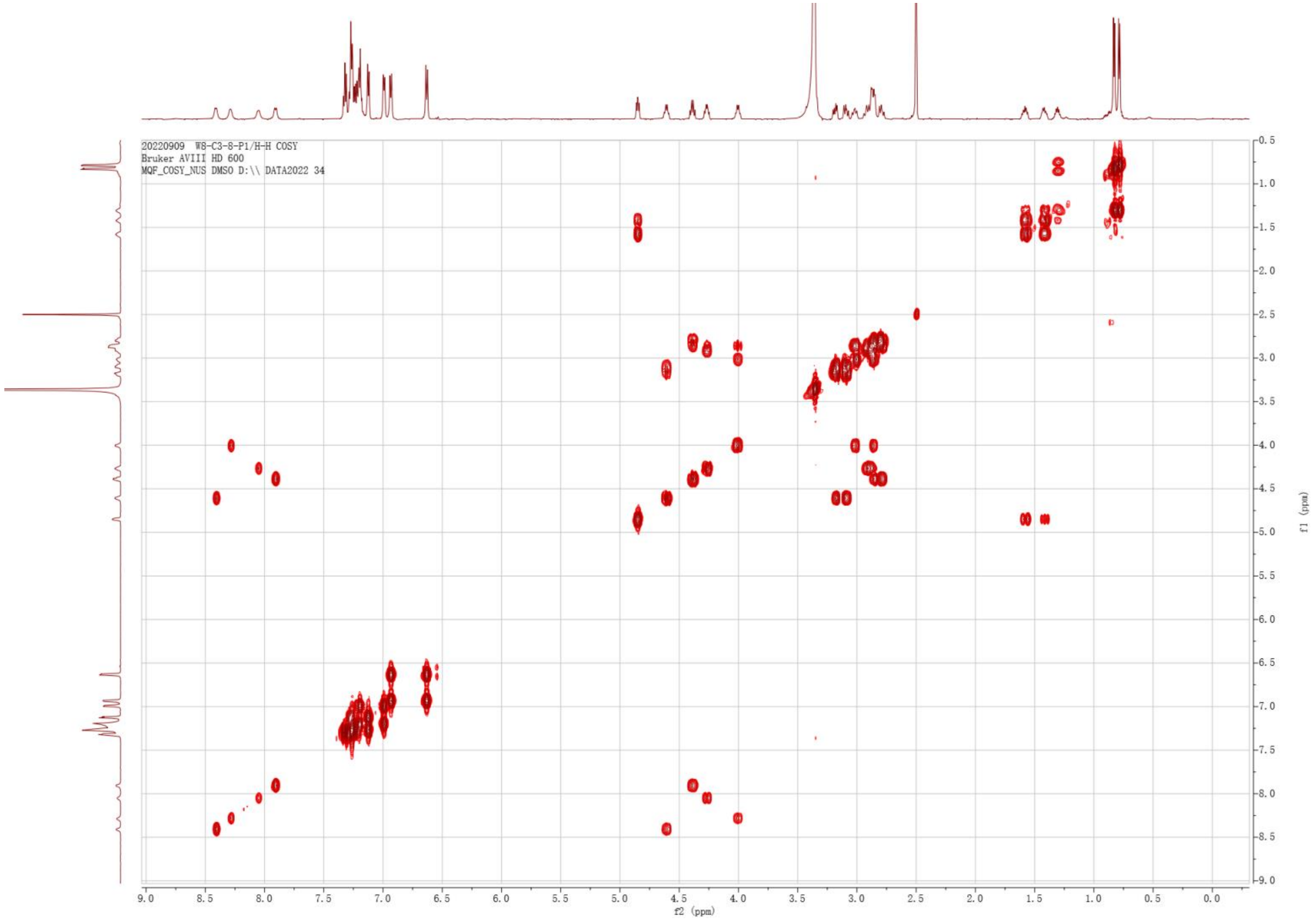

Figure S8. NOESY spectrum of compound 1 at 600 MHz in DMSO-d_6

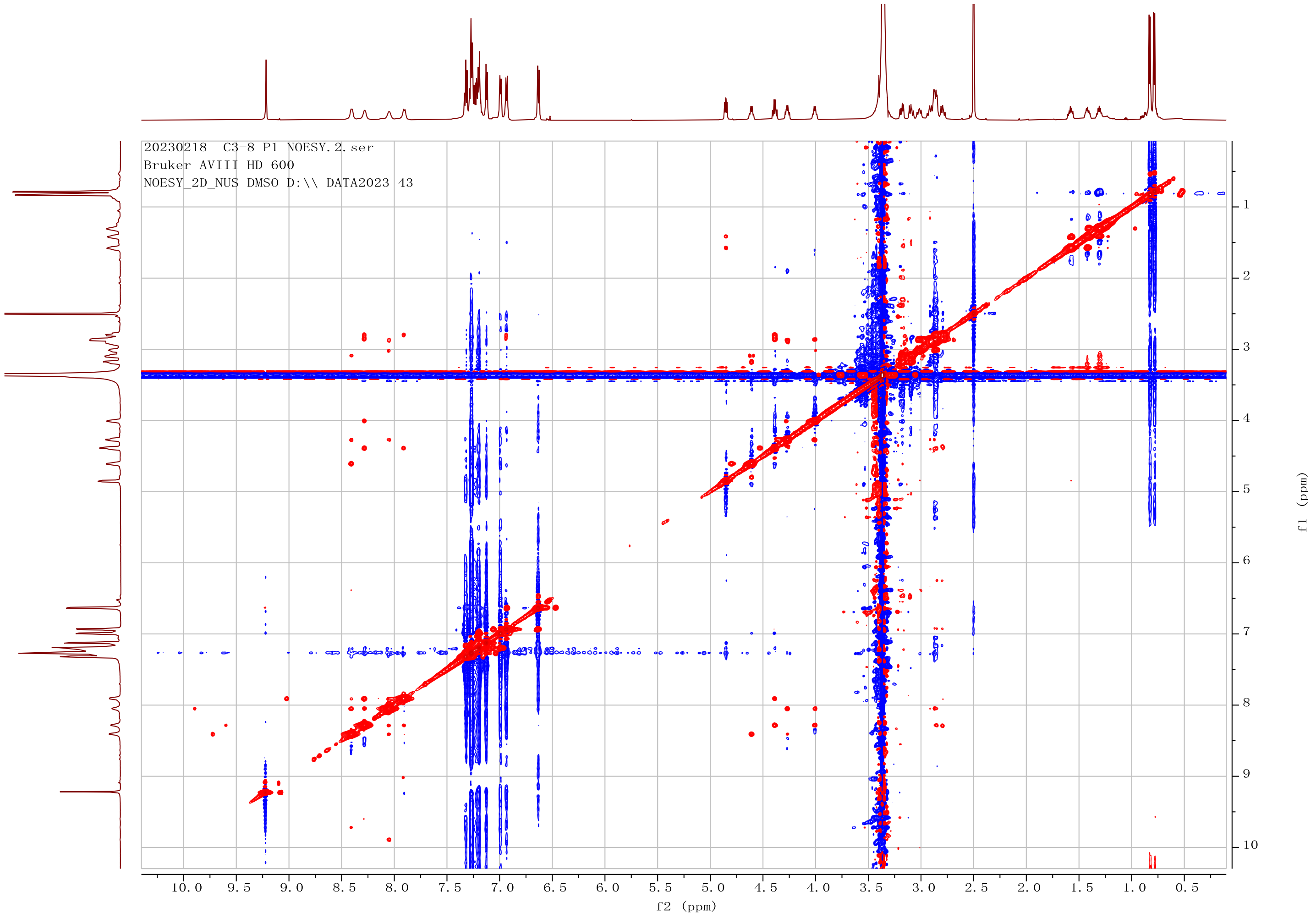

Figure S9. HRESI-MS spectrum of compound 1

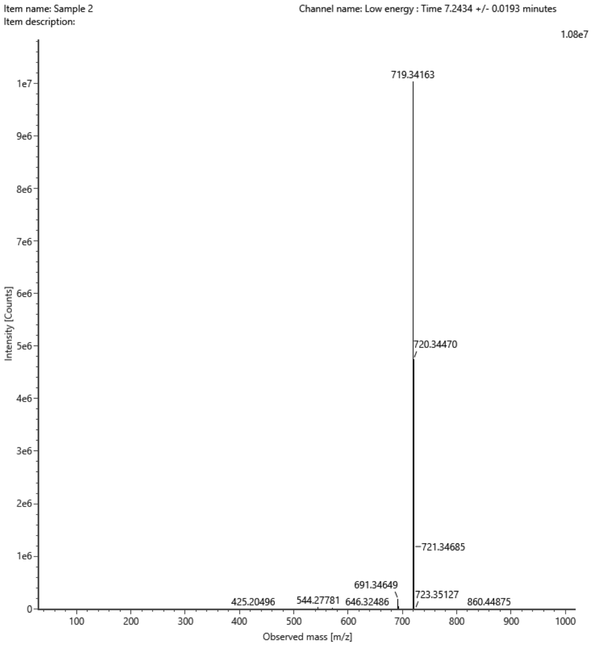

Idx	Mass	Formula	Delta mmu
1	719.34163	$C_{42}H_{47}N_4O_7$	-2.3

Figure S10. HPLC chart of the hydrolysate of compound 1 and the standards(*R*)/(*S*)-Leucic acid

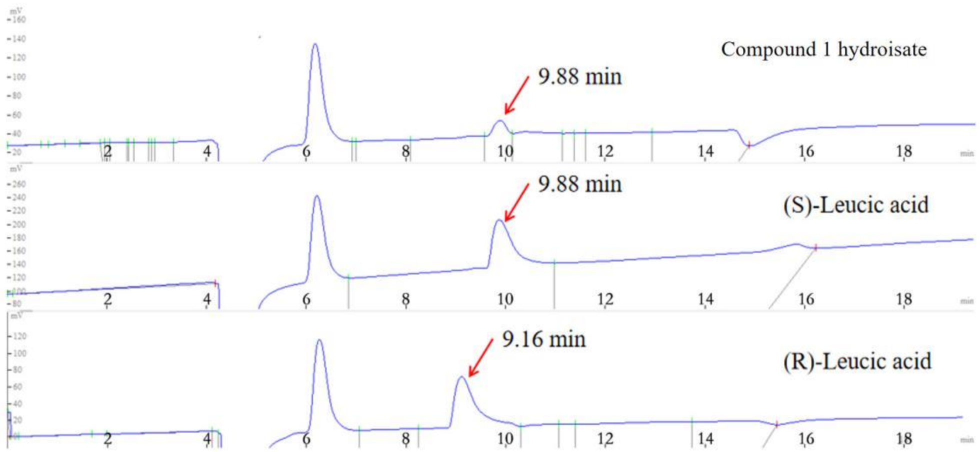

Figure S11. LC-MS analysis of the Marfey' s products of compound 1 and amino acid standards

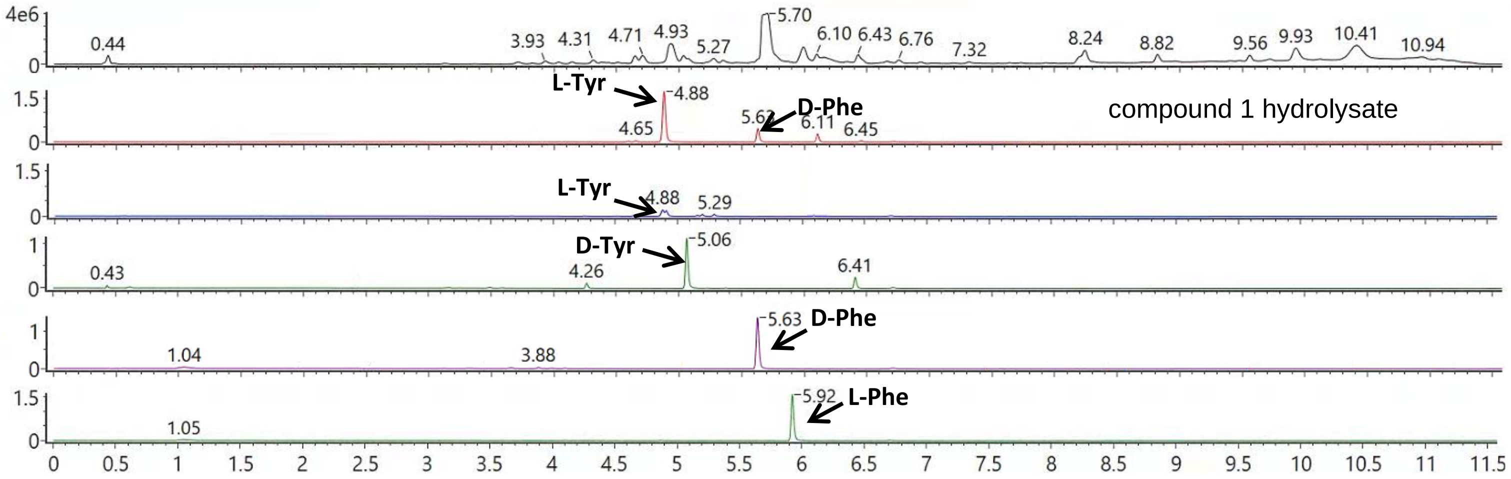

Figure S12. ^{1}H NMR spectrum of compound 2 at 600 MHz in DMSO-d_6

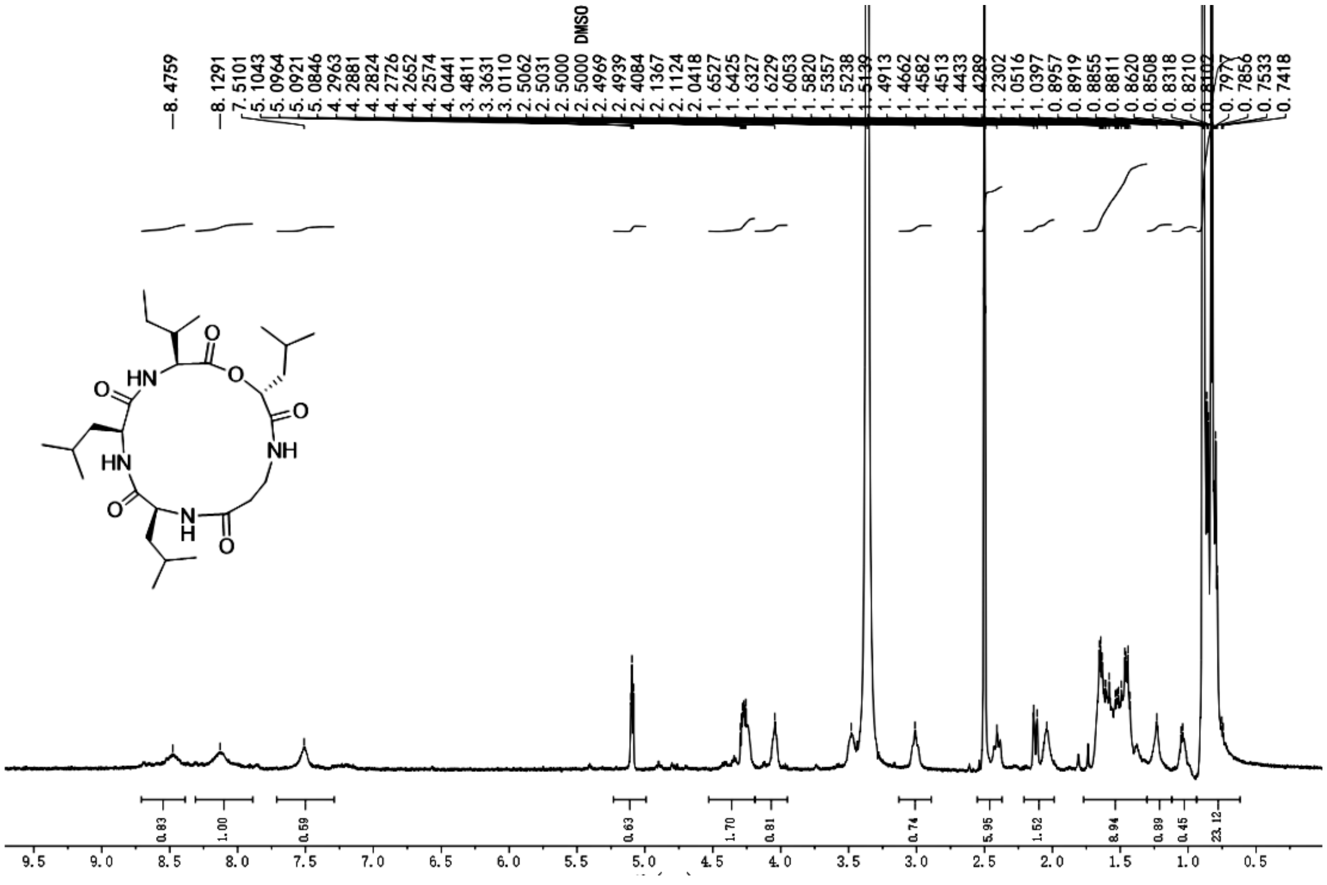

Figure S13. ^{13}C NMR spectrum of compound 2 at 150 MHz in DMSO-d_6

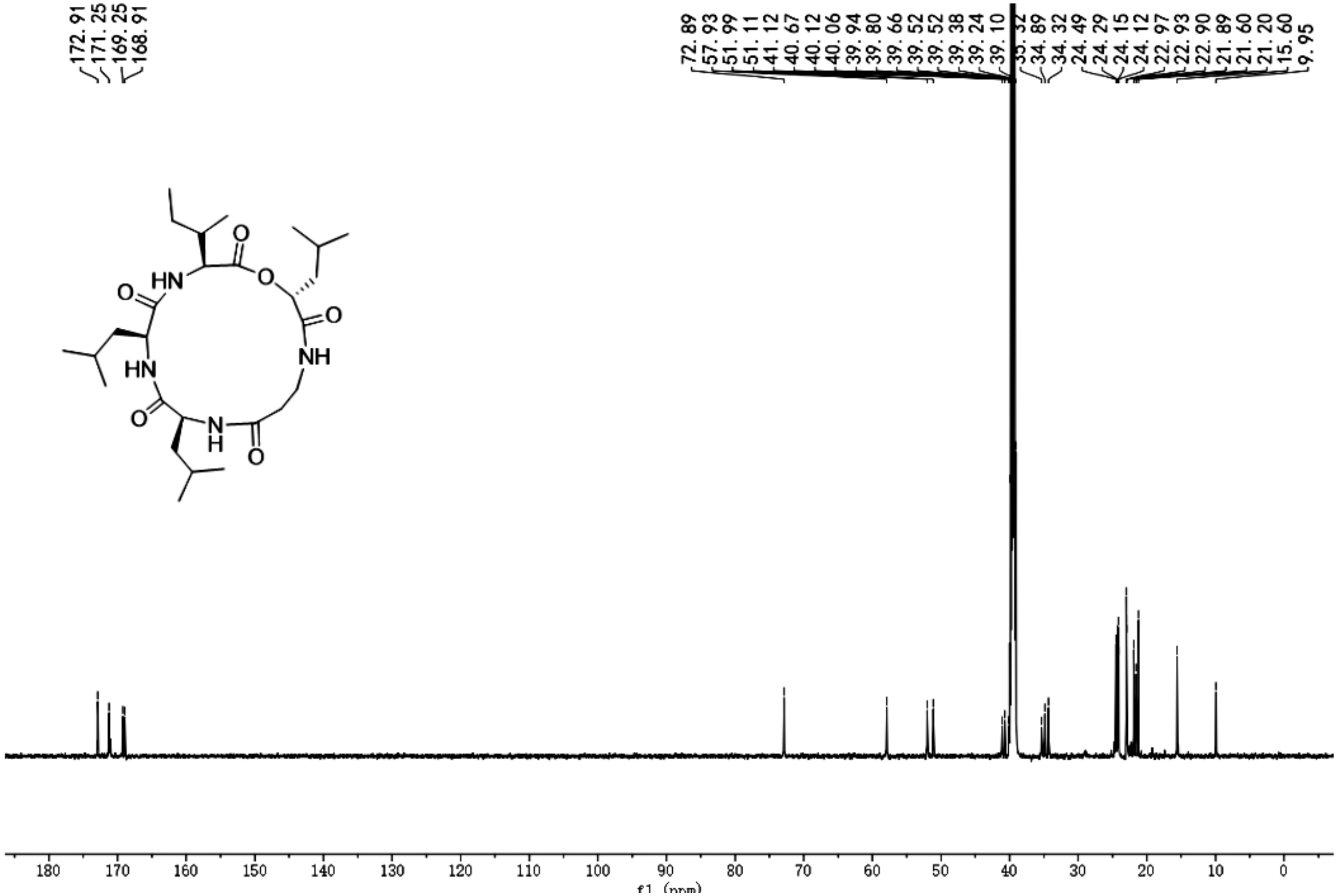

Figure S14. HSQC spectrum of compound 2 at 600 MHz in DMSO-d_6

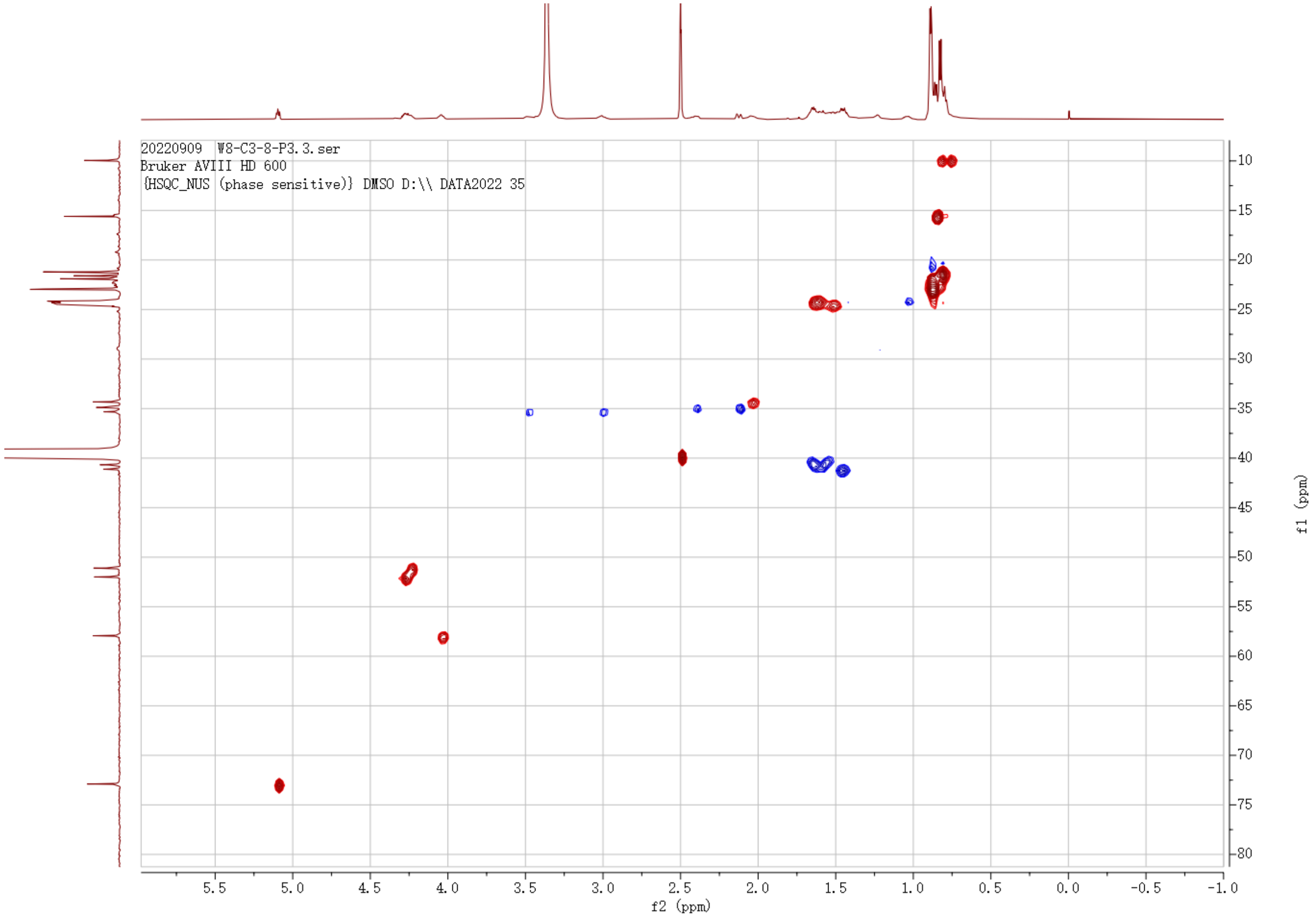

Figure S15. HMBC spectrum of compound 2 at 600 MHz in DMSO-d_6

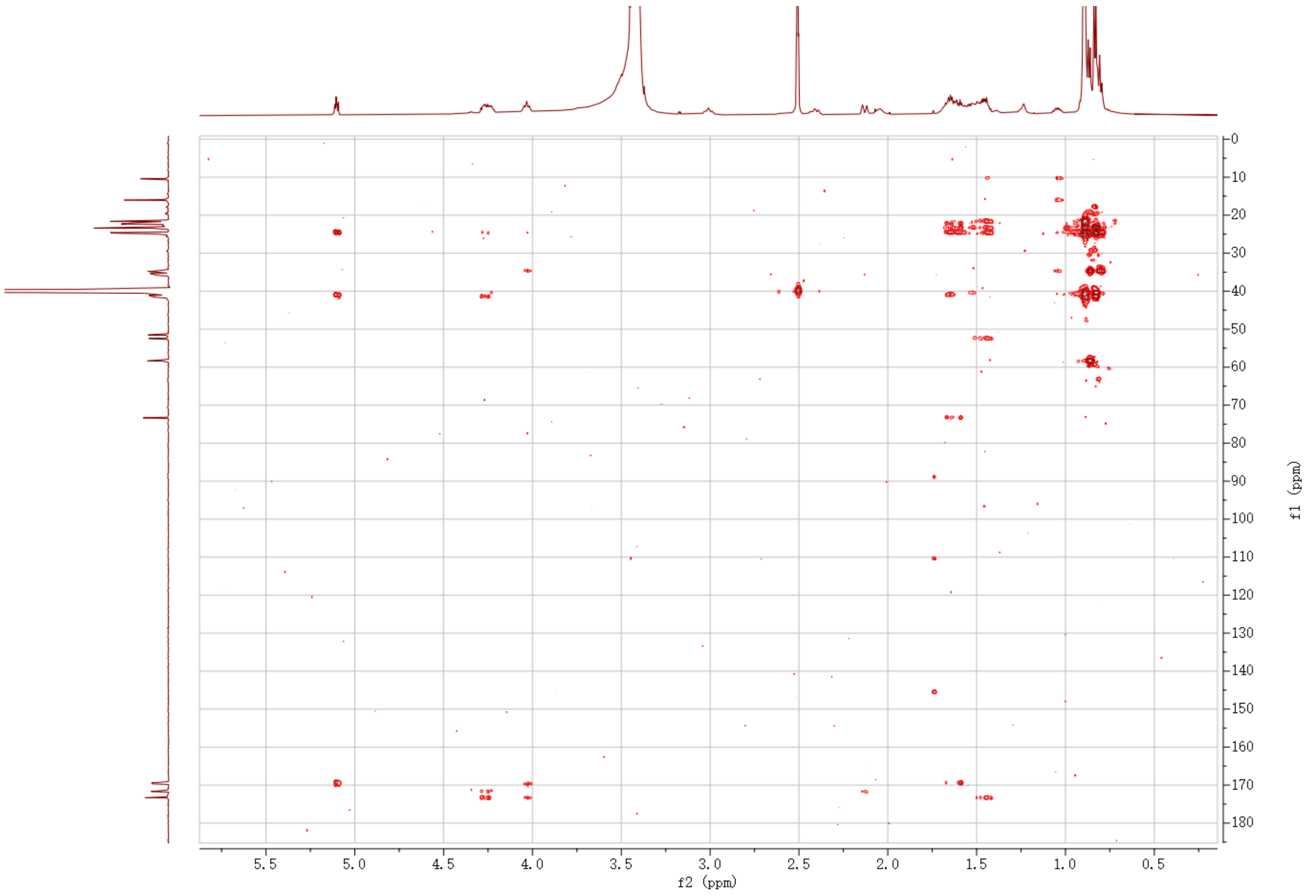

Figure S16. ^{1}H-^{1}H COSY spectrum of compound 2 at 600 MHz in DMSO-d_6

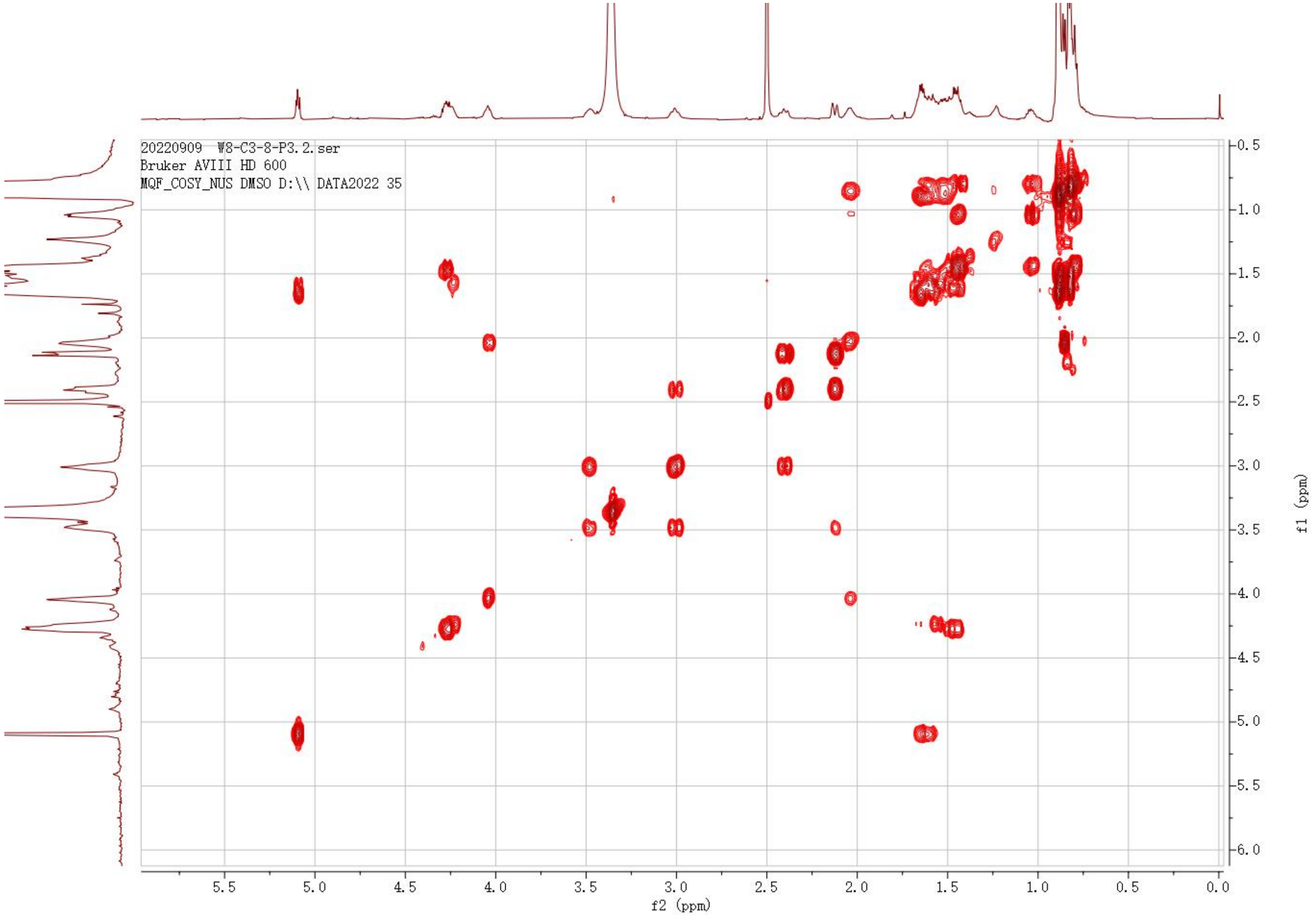

Figure S17. TOCSY spectrum of compound 2 at 600 MHz in DMSO-d_6

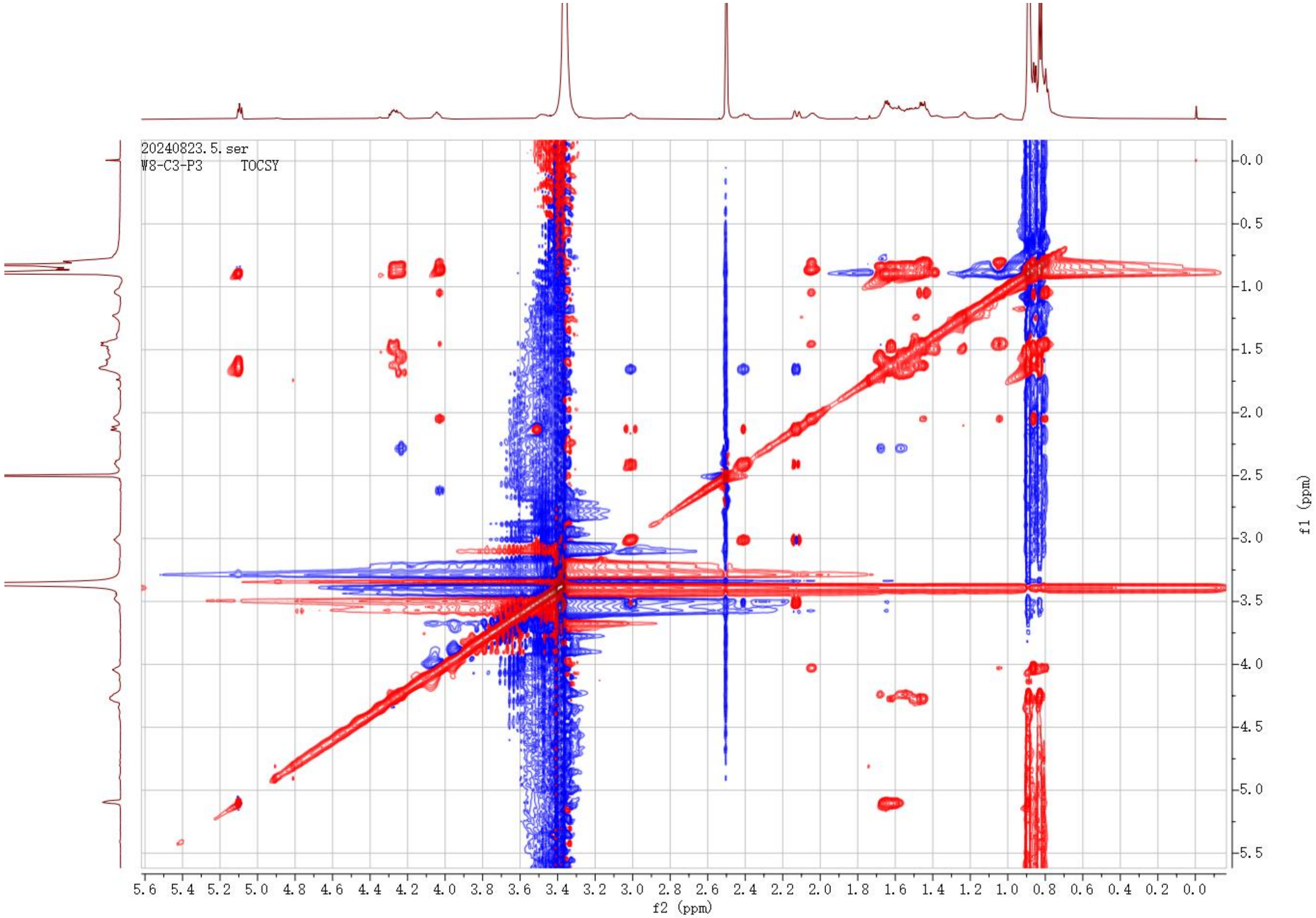

Figure S18. NOESY spectrum of compound 2 at 600 MHz in DMSO-d_6

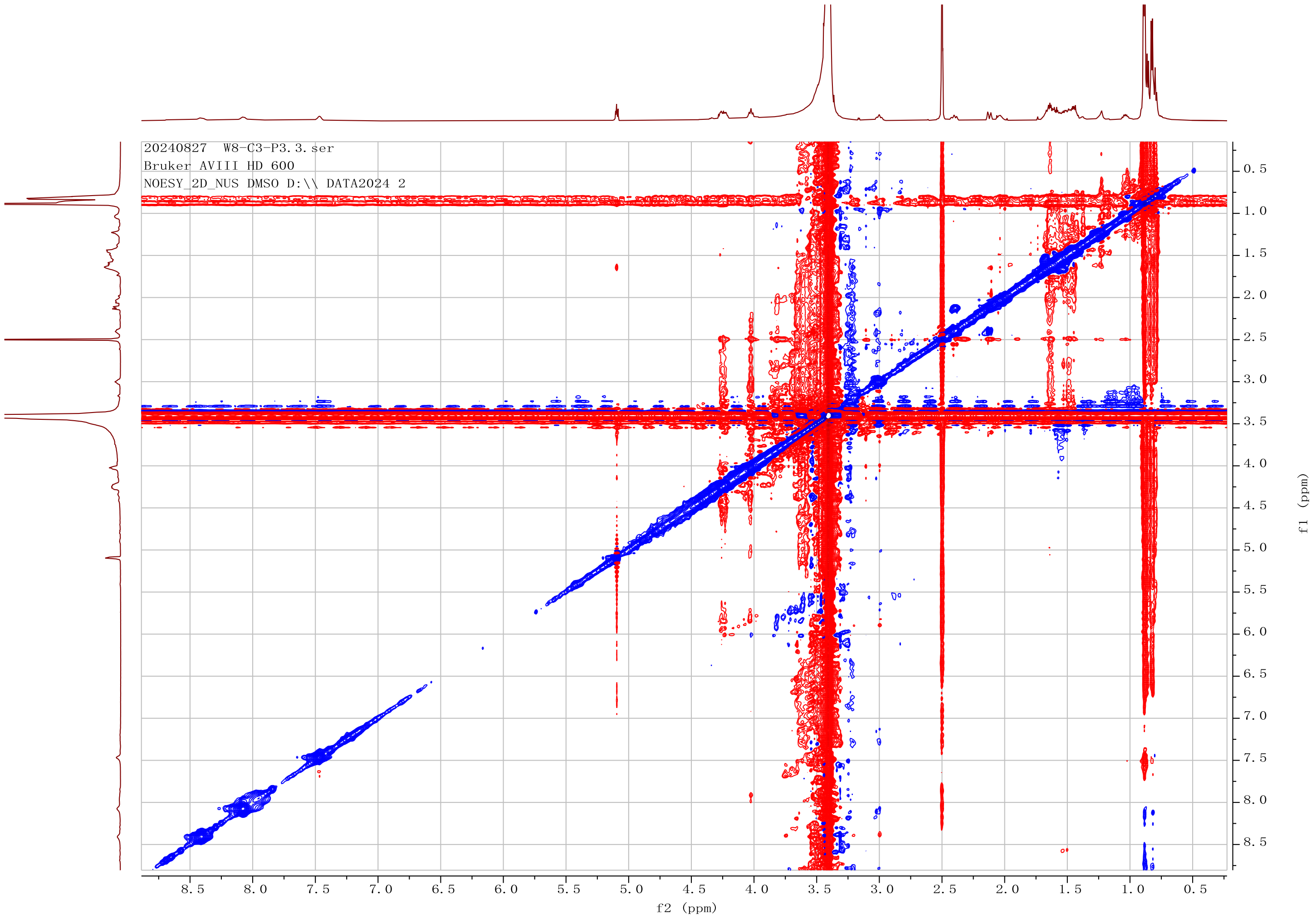

Figure S19. HRESI-MS spectrum of Compound 2

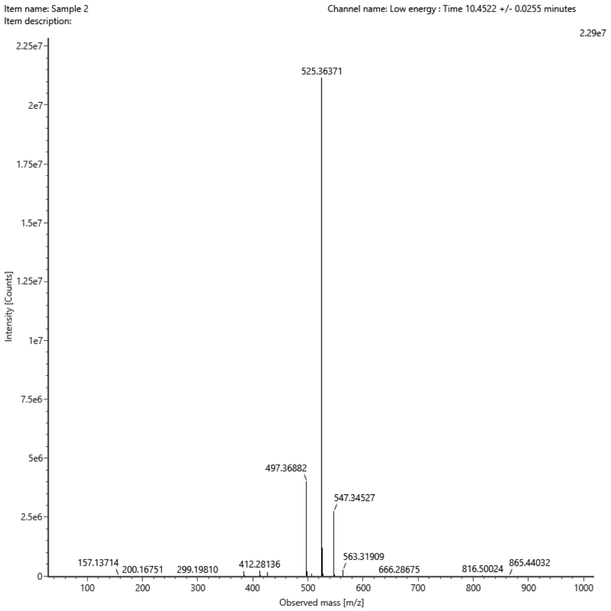

Idx	Mass	Formula	Delta mmu
2	525.36371	$C_{27}H_{48}N_4O_6$	-0.9

Figure S20. HPLC chart of the hydrolysate of compound 2 and the standards(*R*)/(*S*)-Leucic acid

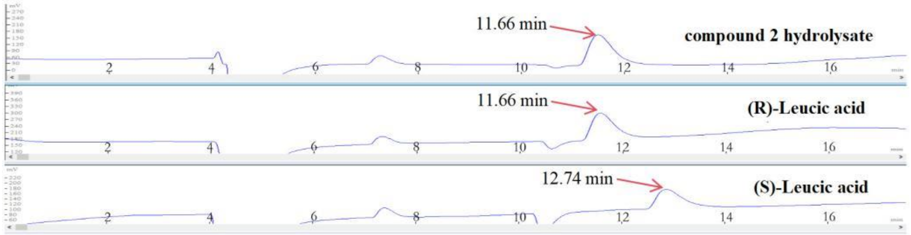

Figure S21. LC-MS analysis of the Marfey' s products of compound 2 and amino acid standards

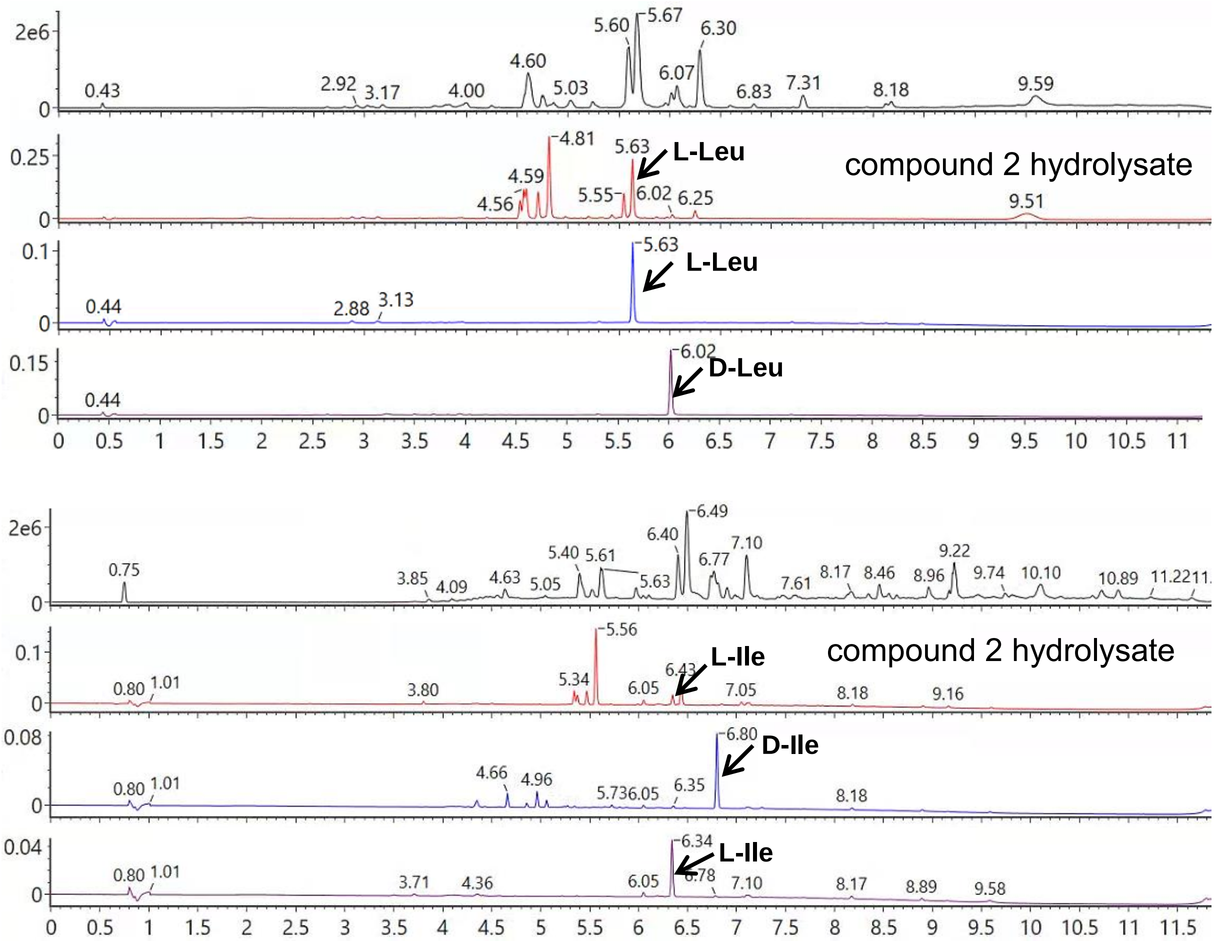

Supplement: Supplementary file 1 [file molecules-29-05746-s001.zip › molecules-3312446-supplementary.pdf]
